# Development of a Cerium-Doped Titanate/Polyphenol Nanostructured Coating for Titanium Implants: Enhancing Antibacterial Properties through Ciprofloxacin Release

**DOI:** 10.1021/acsami.6c03707

**Published:** 2026-03-17

**Authors:** Marcel Jakubowski, Mateusz Hegmit, Maria Ratajczak, Marta Trzaskowska, Aleksandra Maciejczyk, Agata Przekora, Monika Zielińska, Łukasz Ławniczak, Silvia Spriano, Mariusz Sandomierski

**Affiliations:** † Institute of Chemical Technology and Engineering, 49632 Poznan University of Technology, ul. Berdychowo 4, 60-965 Poznań, Poland; ‡ Institute of Building Engineering, 49632 Poznan University of Technology, ul. Piotrowo 5, 60-965 Poznań, Poland; § Department of Tissue Engineering and Regenerative Medicine, 49554 Medical University of Lublin, Chodzki 1, 20-093 Lublin, Poland; ∥ DISAT Department, 19032 Politecnico di Torino, Corso Duca degli Abruzzi 24, 10129 Torino, Italy

**Keywords:** titanium implants, polyphenols, drug delivery, nanostructured coating, ciprofloxacin

## Abstract

Titanium implants are widely used in orthopedics and dentistry; however, their long-term performance is challenged by bacterial colonization, lack of antioxidant activity, and mismatch in mechanical properties with bone tissue. In this study, we developed a hybrid nanostructured coating composed of cerium-doped titanate and a poly­(tannic acid) layer, further functionalized with ciprofloxacin to enhance antibacterial properties. The coating was synthesized through sequential hydrothermal, ion-exchange, and polyphenol deposition steps, followed by antibiotic loading. Comprehensive characterization using SEM, AFM, XPS, FT-IR, and nanoindentation confirmed the successful formation of a uniform nanostructured coating with drug adsorption capability. Antioxidant activity studies demonstrated the material’s ability to scavenge both free radicals and hydrogen peroxide. The obtained materials also exhibited the ability to neutralize intracellular reactive oxygen species (ROS). The scavenging of free radicals was facilitated by the polyphenolic component of the coating, whereas the decomposition of H_2_O_2_ was enabled by the presence of Ce^3+^ ions. Drug release experiments showed controlled ciprofloxacin release for up to 34 h, ensuring effective antibacterial action during the critical early stage after implantation. In vitro studies with osteoblasts confirmed the biocompatibility of the coatings, while antibacterial tests against *Staphylococcus aureus* and *Pseudomonas aeruginosa* revealed almost complete inhibition of bacterial growth. Importantly, the modified surface exhibited a Young’s modulus closer to that of human bone, suggesting a potential reduction of the stress shielding effect. Overall, the developed cerium-titanate/polyphenol/antibiotic hybrid nanostructured coating represents a promising strategy to improve the performance and safety of titanium-based implants.

## Graphical abstract

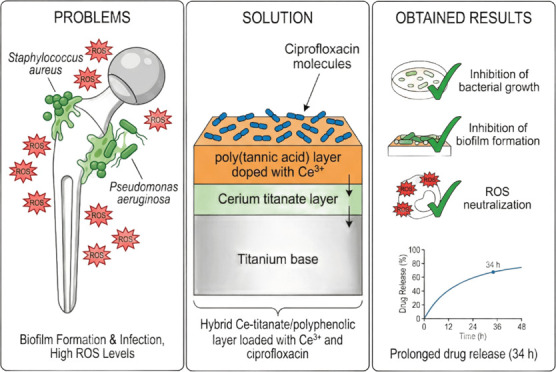

## 1 Introduction

Titanium and its alloys (e.g., Ti_6_Al_4_V) are among the most commonly used materials for orthopedic and dental implants. This is due to several reasons. First, these materials possess excellent mechanical properties; for example, the Ti_6_Al_4_V alloy has a tensile strength of approximately 940 MPa. Second, they exhibit bioinertness, meaning they do not cause allergic reactions in the body after implantation.^1^ Additionally, titanium alloys have high corrosion resistance.^2^ However, the use of titanium for implants also has certain drawbacks. The most significant one is the potential formation of bacterial biofilms on their surface. This can lead to bacterial infections after implantation, which cannot be treated with oral or intravenous antibiotics, as biofilms often exhibit resistance to them.^3^ As a result, a second surgery to remove the implant may be necessary, posing a significant risk to the health and life of patients.^4^ Another issue related to implants is the relatively long osseointegration time. Although titanium does not cause allergic reactions, it is still recognized by the body as a foreign object, which cells attempt to isolate by enclosing it in a fibrotic capsule.^5^ Some reports in the literature indicate that one of the factors slowing down osteogenesis is mitochondrial damage caused by the production of reactive oxygen species (ROS) by cells in response to the body’s inflammatory reaction.^6^ The speed of the osseointegration process is particularly crucial in the case of a poor quality of the bone and dental implants. This is because the oral cavity contains a large number of microorganisms that can migrate to the implant area and cause an infection. Also studies conducted by various researchers have shown that despite titanium’s excellent corrosion resistance, it undergoes corrosion processes over time after implantation, mainly if micromovements can occur (fretting), releasing titanium, vanadium, and aluminum ions.^7^ One of the additional challenges associated with implants is the stress shielding effect. This phenomenon arises from significant differences in the Young’s modulus between the implant and the surrounding bone, leading to reduced mechanical stimulation of the bone. As a result, it can cause bone resorption and, ultimately, bone loss.^8^ Research on titanium implants focuses on solving the aforementioned problems. For this reason, their surface is subjected to various modifications. One type of modification involves the application of nanostructured coatings.^9^

Nanostructured coatings are characterized by nanoscale surface roughness, which can significantly influence the biological interactions at the implant–bone interface. These coatings enhance the biological response of the material by promoting osteogenic differentiation and supporting more effective osseointegration. In addition, surfaces with nanostructured roughness have been shown to inhibit bacterial adhesion, providing an intrinsic antibacterial effect that helps reduce the risk of biofilm formation. Another key advantage of nanostructured coatings is their positive impact on protein adsorption, which is essential for initiating favorable cellular responses and improving tissue integration. Overall, nanostructured surfaces have emerged as a promising strategy in both biomedical and industrial applications, offering the dual benefits of enhanced biocompatibility and resistance to bacterial colonization.^10^

Such coatings can be obtained in various ways using physical or chemical methods.^11,12^ One of the chemical methods for producing nanotextured coatings on the surface of titanium alloys is the hydrothermal reaction in a sodium hydroxide solution.^13^ This process allows for the formation of a bioactive sodium titanate layer. An additional advantage of these layers is their ion-exchange capability, meaning that during coating preparation sodium ions can be replaced with other ions that significantly influence the response of bone cells, such as calcium or magnesium.^14^ Titanate layers are characterized by their ability to support hydroxyapatite growth, as well as cell adhesion and proliferation.^15^

Another type of modification that enables the formation of functional coatings is the surface modification of titanium using polyphenols.^16^ Polyphenols are natural plant-derived compounds with numerous health-promoting effects. They exhibit strong antioxidant activity and, to some extent, the ability to inhibit bacterial cell growth. They also have numerous biomedical applications. For example, Ghosh et al. used a natural polyphenol, quercetin, as a potential agent for the treatment of corneal dryness.^17^ One compound that belongs to this class of compounds is tannic acid. This polyphenol is highly water-soluble and possesses antibacterial properties, as well as the ability to interact with various metal ions. The ability to interact with metal ions is important, as it allows for additional functionalization of the coating with bioactive ions.^18^ The ions that can be used for this purpose are Ce^3+^ ions. Materials obtained using cerium ions exhibit various functional properties. In terms of bone tissue engineering for example, they can influence osteogenic differentiation and angiogenesis. Some reports in the literature also indicate their ability to inhibit bacterial cell growth. However, the most important properties of cerium ions arise from the unique redox pair Ce^3+^/Ce^4+^.^19^ Owing to this, cerium ions can act as antioxidants and therefore contribute to the inhibition of inflammation. This is an important feature, as an implant recognized by the human body as a foreign object may induce chronic inflammatory responses at the implantation site. Materials containing cerium ions in their structure have previously been used in the development of biomaterials. Yang et al. utilized ceria-based nanocages modified with Poly­(l-Histidine) as a material for the treatment of chemical eye injury.^20^ In another study, Luo et al. employed mesoporous ceria nanoparticles as a carrier for Y-27632, which is used in the treatment of ocular hypertension.^21^ Other studies have also demonstrated the potential of ceria to serve as a drug delivery carrier, for example, for gabapentin.^22^ This also demonstrates the high potential of cerium ions in the development of advanced biomaterials.

Numerous studies have shown that despite certain antibacterial properties, titanate or polyphenol layers alone are too weak to completely inhibit microbial growth.^23^ A currently applied solution to this problem is the use of antibiotics in implant surface modification. Ciprofloxacin (Cipro), is an antibiotic from the fluoroquinolone group known for its strong antibacterial activity. Additionally, it is used in the treatment of bone infections. For this reason, it appears to be an ideal candidate for use in implant surface modification. Ciprofloxacin also has the ability to form complex compounds with various metal ions.^24^

Based on the above considerations, in this study we decided to prepare a coating composed of the components mentioned above, aiming to achieve their synergistic effects and obtain a multifunctional, bioactive surface layer. First, the material was etched in NaOH, which enabled the formation of a nanostructured titanate layer. This layer was then subjected to ion exchange with Ce^3+^. Incorporating Ce^3+^ ions into the titanate structure was expected to facilitate the effective attachment of a tannic-acid–based coating through interactions between the catechol groups and the Ce^3+^ ions. Subsequently, a tannic acid layer was formed via self-oxidative polymerization, and this layer was additionally loaded with Ce^3+^ ions. The resulting system should exhibit the ability to stabilize reactive oxygen species. Moreover, the presence of Ce^3+^ ions both in titanate and polyphenolic parts of the layer was also intended to enable the synergistic loading of ciprofloxacin through coordination interactions. The overall study concept and the layer preparation scheme are presented in Figure 1. Based on literature, the use of a polyphenolic layer for drug delivery is still an emerging approach, with only a limited number of studies exploring its potential.

**Figure 1:**
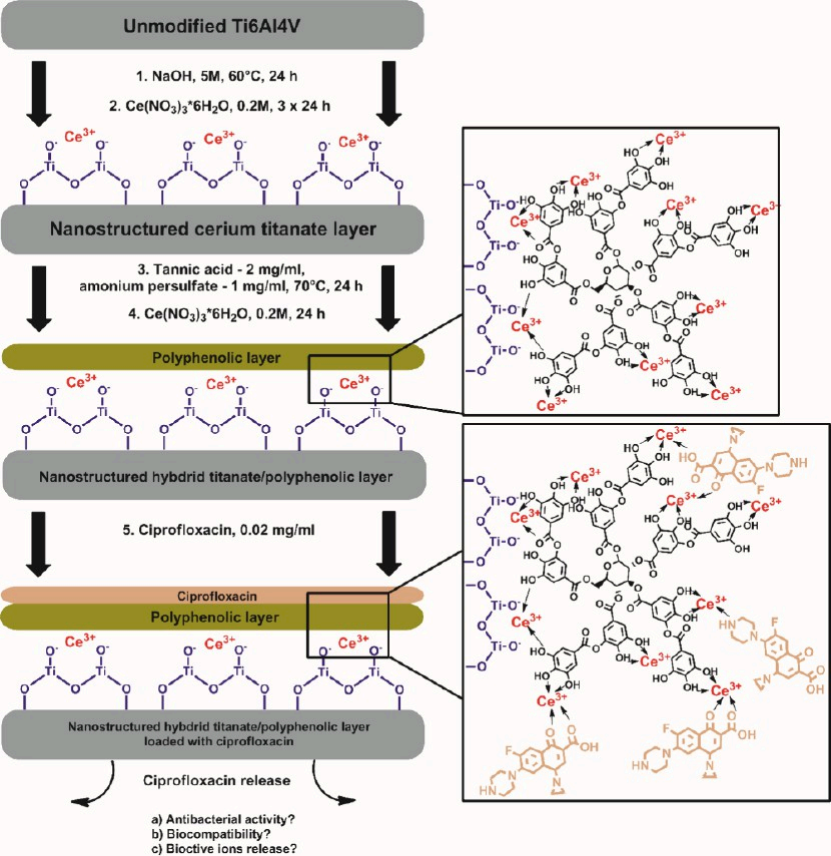
Scheme of the conducted surface modification.

It is also worth emphasizing that one of the risks associated with the use of antibiotics is the potential development of antibiotic resistance in bacterial cells. The problem of microbial antibiotic resistance has been formally acknowledged by the European Union, which in its resolution (2023/2703­(RSP)) of 1 June 2023 identified antimicrobial resistance as one of the three most serious health threats within the EU. As part of its strategy to combat this growing issue, the European Union called on Member States to implement appropriate national measures aimed at reducing the total consumption of antibiotics in humans by 20% by the year 2030, compared to the 2019 baseline. This initiative reflects the urgent need to limit the overuse of antibiotics and promote alternative approaches to preventing and managing infections. Patients undergoing implantation procedures are typically administered prophylactic antibiotics to prevent postoperative infections. However, this approach could be significantly improved or even replaced by enabling the local release of the antibiotic directly at the implantation site. When antibiotics are taken orally, much higher doses are required to ensure that a therapeutic concentration reaches the implantation area. In contrast, controlled release from the implant surface would deliver the drug precisely where it is needed, using far smaller amounts. Such a strategy not only enhances treatment efficiency but also reduces the overall antibiotic burden on the patient. Consequently, it may contribute to lowering the widespread use of antibiotics and, in the long term, help mitigate the growing problem of antimicrobial resistance.

The materials prepared in this study were characterized using various analytical techniques, including scanning electron microscopy (SEM), atomic force microscopy (AFM), and X-ray photoelectron spectroscopy (XPS). Additionally, the hydrophilicity and antioxidant properties of the materials were evaluated. Nanoindentation was used to assess the nanomechanical properties of the prepared nanostructured coating.

## 2 Materials and Methods

### 2.1 Reagents

Tannic acid, sodium hydroxide, cerium nitrate hexahydrate, ammonium persulfate, and ciprofloxacin (pharmaceutical grade) were purchased from Sigma-Aldrich. Acetone and ethanol were obtained from POCH. These reagents were used to prepare the hybrid titanate/polyphenol nanostructured coating. Sodium chloride, sodium bicarbonate, potassium chloride, dipotassium hydrogen phosphate trihydrate, sodium sulfate, TRIS, human serum albumin and HCl were also purchased from Sigma-Aldrich and used for the preparation of simulated body fluid (SBF). 2-diphenyl-1-picrylhydrazyl (DPPH) was used to assess antioxidant properties. Hydrogen peroxide (30% solution in H_2_O), was purchased from Sigma-Aldrich, while the H_2_O_2_ detection kit was obtained from Thermo Fisher Scientific. All reagents were used without further purification.

### 2.2 Titanium Modification with Poly­(tannic acid)/Cerium Titanate Hybrid Nano Layer

To prepare the nanostructured coating, titanium plates (Tit) of 1 cm × 0.8 cm × 0.3 or 1.5 cm × 1.5 cm × 0.3 cm or discs Ø 8 mm and 3 mm thickness were first polished using sandpapers of grits #150, #400, #800, #1200, and #2000. The specimens were then degreased in acetone, ethanol, and deionized water. Next, a sodium titanate layer was formed on the surface. The titanium samples were placed in a 5 M NaOH solution and subjected to a reaction in a sealed vessel at 60 °C for 24 h.^25^ After this period, the plates were rinsed multiple times with deionized water and dried at 100 °C. The obtained samples were labeled as NaTit. To introduce functional ions, the plates were then immersed in a 0.2 M cerium nitrate solution for 24 h. After this time, the solution was replaced with a fresh portion of 0.2 M cerium nitrate solution, and this process was repeated three times. Chosen concentration of cerium nitrate solution was based on our previous studies.^26^ The plates were then rinsed again and dried at 100 °C. These samples were designated as CeTit.

To create the hybrid titanate/polyphenol nanostructured coating, CeTit samples were immersed in 100 mL of a solution containing tannic acid (2 mg/mL) and ammonium persulfate (1 mg/mL) and reacted in a sealed vessel at 70 °C for 24 h. After this synthesis step, the plates were rinsed (designated as CeTit-Tan) and, without drying, immediately placed in a 0.2 M cerium nitrate solution to allow Ce^3+^ ions to bind to the catechol groups in tannic acid. The plates were then rinsed three times with deionized water and dried at 37 °C over the weekend. The final samples, after cerium ion loading, were labeled CeTit-Tan-Ce. The synthesis scheme is shown in Figure 1.

### 2.3 Titanium Surface Characterization

Scanning electron microscopy (SEM) was employed to examine the surface morphology of the prepared nanostructured coatings at different stages of modification. The observations were carried out using a Vega SEM (Tescan, Czech Republic). Surface topography and roughness parameters were determined using atomic force microscopy (AFM), which provides three-dimensional surface images. An NX10 microscope (Park Systems) was employed, with scanning areas of 60 × 60 μm. The elemental composition of the obtained layer was analyzed using energy-dispersive spectroscopy (EDS, Bruker). Measurements were taken at a minimum of ten distinct points, and the results were averaged. XPS analyses were carried out using a PHI VersaProbe II Scanning XPS system with monochromatic Al Kα radiation (1486.6 eV). A 100 μm X-ray beam scanned an area of 400 × 400 μm. Spectra were collected at a 45° takeoff angle, with pass energies of 117.5 eV (0.5 eV step) for survey scans and 46.95 eV (0.1 eV step) for high-resolution scans of the C 1s, O 1s, N 1s, Ti 2p, Ce 3d, and F 1s regions. Dual-beam charge compensation (7 eV Ar^+^ ions and 1 eV electrons) was applied to stabilize the surface potential. Spectra were referenced to the C 1s peak at 285.0 eV, with a chamber pressure below 3.5 × 10^–9^ mbar. Data were processed using PHI MultiPak (v.9.9.3), and background subtraction was performed using the Shirley method. The modification efficiency was further assessed via Fourier-transform infrared spectroscopy/microscopy (FT-IR) in reflection mode, using a gold mirror as the background and a TE-MCT detector, allowing evaluation of layer uniformity and surface drug presence. Surface hydrophilicity was evaluated through water contact angle measurements using an optical tensiometer. Samples were placed on a leveled surface, and image recording was initiated via the software. A drop of distilled water (≈5 μL), was deposited onto the sample, and recording continued for 30 s. The first image capturing the fully settled droplet was used for measurement, and contact angles were calculated with Attension Theta software. For each modification, at least five samples were measured, and the results were averaged. Nanomechanical properties were assessed using a Nanomechanics iMicro nanoindenter with a standard Berkovich tip. Prior to testing, the indenter was calibrated using a quartz glass reference sample. Each indentation was performed until either a maximum force of 50 nN or a depth of 5000 nm was reached, followed by a 1-s hold at the maximum force. Approximately 30 measurements were performed per sample, with the most extreme values discarded to retain 25 valid results.

### 2.4 Drug Sorption and Release

The drug adsorption procedure was carried out using CeTit-Tan-Ce samples with dimensions of 1 cm × 0.8 cm × 0.3 cm. Each sample was individually placed in a 2 mL Eppendorf tube, which was subsequently filled with 2 mL of Cipro solution at a concentration of 0.02 mg/mL. To facilitate efficient adsorption, the tubes were incubated on an orbital shaker at 150 rpm for a duration of 72 h, allowing for continuous and uniform interaction between the drug molecules and the modified titanium surfaces. Following the adsorption period, the remaining solution was analyzed using UV–vis spectroscopy over a wavelength range of 250–400 nm, with the maximum absorbance observed at 279 nm. The amount of Cipro adsorbed onto the surfaces was determined quantitatively through comparison with a pre-established standard calibration curve. To ensure reproducibility and reliability of the data, six independent replicates were conducted for each experimental condition. At the conclusion of this step, the drug-loaded samples were designated as CeTit-Tan-Ce-Cipro.

For the subsequent drug release studies, the Cipro -loaded titanium samples were carefully transferred to 2 mL glass vials, each containing 1 mL of simulated body fluid (SBF) to mimic physiological conditions. The vials were maintained under constant agitation on an orbital shaker set at 150 rpm and a controlled temperature of 37 °C to replicate the dynamic environment of the human body. Samples (1 mL), were collected at predetermined time intervals to monitor the release profile of ciprofloxacin, and new portion of fresh SBF was added after each sampling to maintain consistent experimental conditions. The released drug concentration was quantified using UV–vis spectroscopy, with calibration curves specifically prepared in SBF to account for potential matrix effects.

The amount of drug released into the solution was determined using the eq 1:

$$\mathrm{Drug}\;\mathrm{release}[\mathrm{\mu g}]=C_{t}\times V_{\mathrm{SBF}}\times 1000$$ 1

where *C_t_* [mg/mL] is the concentration of ciprofloxacin calculated from the calibration curve in the solution at a given time, and *V*_SBF_ [mL] is the volume of the release medium. The cumulative release profile was obtained by sequentially summing the amounts released at each measurement point.

Each release experiment was performed in triplicate to ensure statistical significance. The composition of the SBF employed in these studies is summarized in Table 1, providing a physiologically relevant ionic environment for evaluating the release behavior.

**Table 1:** Composition of Simulated Body Fluids per 1000 mL of Solution

order	reagent	amount
1.	NaCl	8.035 g
2.	NaHCO_3_	0.355 g
3.	KCl	0.225 g
4.	K_2_HPO_4_·3H_2_O	0.231 g
5.	Na_2_SO_4_	0.072 g
6.	TRIS	0.6112 g
7.	HCl	0–5 mL

Additionally, to further replicate the conditions present in the human body, the release study was also carried out in SBF enriched with human serum albumin at a concentration of 1 mg/mL. The experimental procedure was identical to that used for samples in which release was carried out in conventional SBF with the exception that drug release was analyzed using HPLC–UV/DAD. Cipro release was quantified using an Agilent 1260 Infinity II HPLC system equipped with a quaternary pump (600 bar) and a DAD detector. Chromatographic separation was performed on a Zorbax Eclipse XDB-C18 column (150 × 4.6 mm, 5 μm), maintained at 30 °C. The mobile phase consisted of 10 mM potassium dihydrogen phosphate buffer adjusted to pH 3.0 (solvent A) and acetonitrile (solvent B). The analysis was carried out in gradient mode as follows: 20% B was maintained for the initial 5 min, then increased linearly to 70% B over 20 min, followed by a 10 min re-equilibration step back to the initial conditions. The flow rate was set at 0.8 mL/min. Detection was conducted at 279 nm, with an injection volume of 10 μL. A calibration curve for CIPRO was prepared in the concentration range of 0.02–20 μg/mL, showing good linearity with a correlation coefficient (*R* ^2^) of 0.999. The concentration of CIPRO released was determined by interpolation from the calibration curve. The limits of detection (LOD) and quantification (LOQ) were 0.016 and 0.047 μg/mL, respectively. Each data point represents the mean value of three independent samples, each analyzed in duplicate.

### 2.5 Antioxidant Properties

The DPPH assay was employed to assess the antioxidant capacity of the tested materials, specifically their ability to scavenge the stable free radical 2,2-diphenyl-1-picrylhydrazyl (DPPH). The experimental procedure followed has been previously described in the literature.^6^ Initially, a DPPH solution was prepared in methanol at a concentration of 0.024 mg/mL. Titanium plates with dimensions of 1 cm × 0.8 cm × 0.3 cm were then placed into individual glass vials, and 1 mL of the freshly prepared DPPH solution (initial absorbance at 517 nm = 0.576) was added to each vial. A vial containing only the DPPH solution served as a control to provide a reference for radical concentration. The samples were incubated in the dark for 30 min to prevent photodegradation of the DPPH radical. After the incubation period, the absorbance of each sample was measured at 517 nm using a UV–vis spectrophotometer. The radical scavenging activity of the materials was calculated according to the following formula [Disp-formula eq2]:

$$\mathrm{DPPH}\;\mathrm{scavenging}\;\mathrm{activity}(\%)=\left[\frac{\left(A_{517\;\mathrm{nm}\;\mathrm{control}}-A_{517\;\mathrm{nm}\;\mathrm{sample}}\right)}{A_{517\;\mathrm{nm}\;\mathrm{control}}}\right]\times 100\%$$ 2

Where: *A*_517 nm control_ represents the absorbance of the control sample solution at a wavelength of 517 nm, *A*_517 nm sample_ represents the absorbance of the sample solution at a wavelength of 517 nm.

The H_2_O_2_ decomposition test was conducted as follows. First, the samples (1 × 0.8 cm × 0.3 cm), were placed in 2 mL vials, and then 1.2 mL of a 150 μM H_2_O_2_ solution was added. The samples were then incubated for 24 h. After this period, the H_2_O_2_ concentration was determined with the help of UV–vis spectroscopy, using the H_2_O_2_ detection kit according to the manufacturer’s instructions. The test was conducted for a minimum of three samples. The percentage of degraded H_2_O_2_ was calculated using the following formula [Disp-formula eq3]:

$$H_{2}O_{2}\;\mathrm{scavenging}\;\mathrm{activity}(\%)=\left[\frac{C_{0}-C_{24\;h}}{C_{0}}\right]\times 100\%$$ 3

### 2.6 Cell Response to the Developed Materials

Human fetal osteoblasts (hFOB 1.19 cell line, CRL-3602) obtained from the American Type Culture Collection (ATCC-LGC Standards, UK) were used in the cell culture experiments. The hFOB cells were maintained at 34 °C in a humidified atmosphere (95%) with 5% carbon dioxide and cultured in 1:1 mixture of Ham’s F12 Medium Dulbecco’s Modified Eagle’s Medium (without phenol red) supplemented with 2.5 mM l-glutamine, 0.3 mg/mL G418 (Sigma-Aldrich Chemicals, Poland), 10% fetal bovine serum (FBS, Pan-Biotech GmbH, Germany), 100 μg/mL streptomycin, and 100 U/mL penicillin (Sigma-Aldrich Chemicals, Poland).

#### 2.6.1 Indirect Cytotoxicity Test

To assess cytotoxicity, hFOB 1.19 cells were seeded in 100 μL of a culture medium at a density of 2 × 10^4^ cells per well in 96-multiwell plates and cultured for 24 h. Next, medium was replaced with the sample extracts prepared according to ISO 10993–5 and 10993–12 standards, by immersion of the sample with a 3 cm^2^ surface area in 1 mL of culture medium for 24 h at 37 °C. The cells were further cultured for 24 h and cell viability was analyzed using the MTT colorimetric assay (Sigma-Aldrich Chemical, Warsaw, Poland) in accordance with ISO 10993–5. The test was performed in three independent replicates, and the results were expressed as a percentage relative to the absorbance of the negative control (cells cultured in polystyrene extract).

#### 2.6.2 Direct Cytotoxicity Test

To evaluate cytotoxicity in direct contact with the samples, hFOB 1.19 cells were seeded in 500 μL of a culture medium at a density of 5 × 10^4^ cells per tested materials positioned in 24-well plates and incubated under standard culture conditions for 72 h. Subsequently, cell viability was assessed using a Live/Dead double fluorescent staining kit (Sigma-Aldrich Chemicals), and samples were analyzed by confocal laser scanning microscopy (CLSM; Olympus Fluoview equipped with FV1000). Viable cells were identified by intense green fluorescence from calcein-AM, whereas dead cells exhibited red fluorescence due to staining with propidium iodide (PI).

### 2.7 Inhibition of Intracellular Reactive Oxygen Species Production

Human osteoblasts (hFOB 1.19) were seeded in black, clear bottom 24-multiwell plates in 600 μL complete culture medium at a concentration of 1 × 10^5^ cells/well. After 24-h incubation at 34 °C the growth medium was gently discarded and cells were loaded with 25 μM 2′,7′-dichloroflurescin diacetate dye (DCF-DA, Sigma-Aldrich Chemicals) solution prepared in complete DMEM/Ham F12 medium (400 μL/well) and incubated for 1 h at 37 °C in the dark. DCF-DA after entering the cells membrane is deacetylated by intracellular esterases to a nonfluorescent compound, which is oxidized by reactive oxygen species (ROS) into green fluorescent DCF that can be detected using fluorescence plate reader or microscopy. Next, the cells were washed with PBS buffer and activated for ROS production by addition of 100 μM H_2_O_2_ (Avantor Performance Materials, Poland) prepared in Hanks̀ Balanced Salt Solution (HBSS, Sigma-Aldrich Chemicals) containing 10% FBS. Tested samples were gently placed on the monolayer of cells, incubated in a dark for 3 h at 37 °C, and fluorescence intensity was measured using microplate reader with the excitation wavelength at 485 nm and emission wavelength at 528 nm. The cells cultured in HBSS containing 10% FBS but without 100 μM H_2_O_2_ served as a negative control, showing physiological level of intracellular ROS. The cells cultured in the presence of 100 μM H_2_O_2_ but without tested biomaterials were a positive control, revealing elevated intracellular ROS production. The experiment was performed in four repetitions.

### 2.8 Antibacterial Tests

#### 2.8.1 Bacteria Growth Conditions

In order to test the antibacterial activity of the materials, the Gram (−) *Pseudomonas aeruginosa* and Gram (+) *Staphylococcus aureus* strains were selected. The bacteria were inoculated onto Muller-Hilton (M-H) agarose medium and then placed in an incubator for 24 h at 37 °C. A single bacterial colony was collected and transferred to Muller-Hinton broth and incubated for another 24 h at 37 °C.

#### 2.8.2 Antibacterial Test in Direct Contact with the Materials

The materials were placed in a 24-well plate and rinsed with M-H broth. Simultaneously, a bacterial suspension was prepared in M-H broth diluted twice with PBS. The samples were then inoculated with 500 μL of the bacterial suspension at a density of 1.5 × 10^5^ CFU/mL. The plates with the biomaterials were placed in an incubator at 37 °C for 24 h. After this time, the wells with the bacterial suspension and the materials were carefully pipetted, 100 μL of the bacterial suspension was removed from each and transferred to a 96-well plate. The optical density (OD) of the bacterial suspension was measured at 600 nm. The OD measurement of bacteria cultured with the material was presented as a percentage of the OD value of the positive control (bacteria cultured without the material). The negative control was sterile, diluted M-H broth.

#### 2.8.3 Agar Diffusion Test

Bacteria suspended in M-H broth were diluted to a density of 0.5 McF. A cotton swab was dipped into the bacterial suspension and spread onto a Petri dish with M-H agar. Materials in the form of 7 mm-diameter disks were then placed on the plates. A 6 mm-diameter disk containing the Cipro (10 μg, Oxoid) was used as a positive control. The zone of growth inhibition was defined as half the diameter of the zone of inhibition minus the diameter of the disk. The result was reported in millimeters.

#### 2.8.4 Antibiofilm Activity

To evaluate the viability of bacteria within the developed biofilm, the tested biomaterials were inoculated with two different bacterial strains. Biofilm growing on polystyrene was used as a control. Biomaterials were placed into a 24-well plate and washed with Mueller–Hinton broth. Subsequently, a 500 μL of bacterial suspension of either *S. aureus* or *P. aeruginosa*, adjusted to 0.5 McFarland in M–H broth, was added to the biomaterials and incubated at 37 °C for 24 h. After incubation, the biomaterials were rinsed with PBS to remove nonadherent bacterial cells. Biofilm-forming bacteria were then stained using a Live/Dead Assay Kit for bacteria (Biotium, CA) following the manufacturer’s instructions. Viable and dead bacteria emitted green fluorescence, while only dead cells with disrupted membranes emitted red fluorescence. Imaging was performed using a confocal laser scanning microscope (CLSM; Olympus Fluoview FV1000, Olympus, Tokyo, Japan).

### 2.9 Statistical Analysis

All statistical analyses were performed using one-way ANOVA to evaluate differences between the tested surface modification variants. Tukey’s post hoc multiple comparison test was applied to identify differences between individual groups. Statistical significance was accepted at *p* < 0.05. All statistical analyses were conducted using GraphPad Prism software (version 10.4.2; GraphPad Software, San Diego, CA, USA).

## 3 Results and Discussion

### 3.1 Material Characterization

First, the morphology of the obtained nanostructured coating was evaluated using scanning electron microscopy (SEM), and the images are presented in Figure 2. The surface of the unmodified Ti_6_Al_4_V alloy appears relatively smooth. After the formation of the cerium titanate layer, the surface becomes noticeably porous and rough. Modification of the CeTit sample with a polyphenol layer derived from tannic acid results in a material with a hybrid titanate-polyphenol nanostructured coating on its surface. As observed in the SEM images, this synthesis step leads to surface smoothing. Additionally, the absence of distinct new crystalline structures suggests the formation of a nanometer-thick coating. After loading the sample, which already contained the tannic acid layer, with additional Ce^3+^ ions, no significant changes in surface morphology were observed. This is a favorable outcome, as it indicates that no cerium nitrate salt precipitated in the form of crystals on the surface. Finally, after loading the samples with Cipro, no substantial morphological alterations were detected, further confirming that ciprofloxacin did not precipitate on the material’s surface but rather interacted with the prepared layer as single molecules.

**Figure 2:**
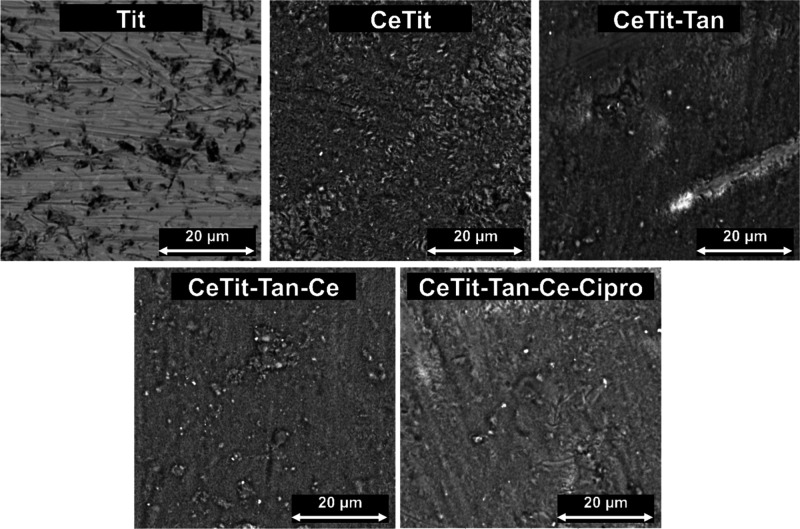
SEM images of the prepared materials at various stages of synthesis.

To further confirm the formation of the hybrid nanostructured coating, atomic force microscopy (AFM) analysis was performed. The results are presented in Figure 3 and Table 2. As observed, the titanium surface is relatively smooth with low roughness (*Sa* value of approximately 75 nm). After the formation of the sodium titanate layer, surface roughness significantly increases, with the Sa rising from around 75 to 137 nm. Surface topography maps also reveal a more porous structure. Following ion exchange with Ce^3+^, the average roughness slightly decreases. The decrease in surface roughness observed after the formation of the polyphenol layer and subsequent loading with trivalent ions may be attributed to the formation of a uniformly distributed thin polymer film on the surface. Additionally, during the loading of the polyphenol layer with trivalent ions, coordination occurs between the metal ions and the phenolic groups of tannic acid, as well documented in the literature.^27,28^ This interaction can induce a slight reorganization of the tannic acid molecules on the surface, leading to a smoother surface morphology. Finally, after ciprofloxacin loading, an increase in roughness is noted, with an *Sq* of 208 nm, and *Sa* of 157 nm. Most likely, ciprofloxacin sorption is a surface-related process. This may be caused by the formation of surface complexes, which in turn can increase the surface roughness. As mentioned in the Section 1, roughness plays a crucial role in the biological response to implants. The AFM results demonstrate that the obtained coating exhibits nanoroughness.^29^ Recent studies suggest that materials with nanoscale roughness have the ability to inhibit bacterial adhesion while promoting better osteoblast attachment.^10,30^ Moreover, the *Sa*/*Sq* ratio is always close to 0.8, which is what is expected for surface features with an almost Gaussian shape. This is important because sharp peaks could negatively affect osteoblast adhesion.^31^ Therefore, it can be concluded that the obtained layer should exhibit enhanced bioactivity and biological response.

**Figure 3:**
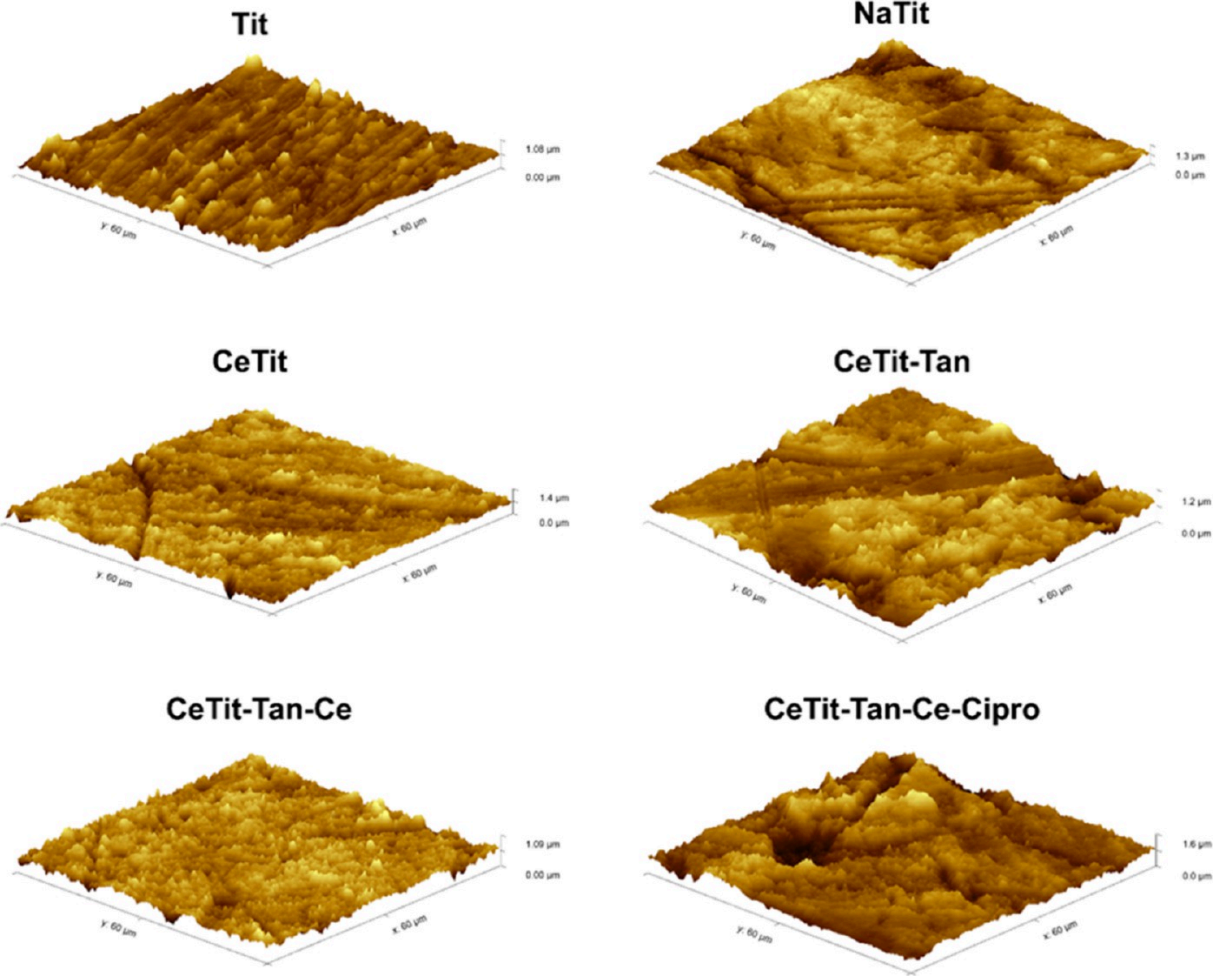
3D AFM images of the material topography at various stages of synthesis, shown for an area of 60 × 60 μm.

**Table 2:** Surface Roughness Parameters (nm) Based on AFM Analysis

	Tit	NaTit	CeTit	CeTit-Tan	CeTit-Tan-Ce	CeTit-Tan-Ce-Cipro
RMS roughness (*Sq*)	100.05	171.31	152.78	145.28	109.56	207.67
mean roughness (*Sa*)	75.31	137.32	119.84	114.41	84.29	156.98

Next, the material were analyzed for elemental composition (EDS). The results are presented in Table 3. As observed, after the synthesis of the titanate layer, the titanium content significantly decreases, while the oxygen content increases. This is associated with the formation of a sodium titanate layer on the surface, which is confirmed by the presence of sodium in the sample.^32^ Additionally, during the treatment of Ti alloys with NaOH, a substantial amount of TiO_2_ forms under the titanate layer. Both this processes explain the high oxygen content.^33^ Following ion exchange with cerium, sodium is completely replaced by Ce^3+^ ions. A similar phenomenon has been reported in other studies.^34^ After the synthesis of the polyphenol layer, the cerium content decreases due to the introduction of new elements, such as carbon from tannic acid, indicating successful modification. Further cerium loading results in more than a 2-fold increase in its content. Finally, after Cipro loading, the cerium content decreases again, which is attributed to the presence of new elements from the ciprofloxacin structure on the surface. All changes in cerium content between consecutive synthesis steps were statistically significant (*p* < 0.05). Additionally, EDS mapping was performed to analyze the distribution of cerium on the surface. All maps (Figure 4a) show a uniform distribution of cerium across the surface. This is a positive outcome, as the even distribution of cerium ions is expected to significantly influence the distribution of the drug after the adsorption process.

**Table 3:** EDS Results for the Elemental Composition of the Obtained Materials (at%)^a^

	Ti	C	O	Al	Na	Ce
Tit	87.6 ± 0.6	2.6 ± 0.5		9.8 ± 0.2		
NaTit	35 ± 2.1^A^	7.8 ± 1.2^A^	51.7 ± 0.7^A^	2.4 ± 0.4^A^	3.2 ± 0.2	
CeTit	33.7 ± 2.3^A^	7.8 ± 1.1^A^	54.5 ± 1.2^AB^	1.5 ± 0.3^AB^		2.5 ± 0.1
CeTit-Tan	33.6 ± 1.5^A^	10.9 ± 1.1^ABC^	52.4 ± 0.7^AC^	1.7 ± 0.3^AB^		1.4 ± 0.12^C^
CeTit-Tan-Ce	31.8 ± 1.5^AB^	14.9 ± 2.7^ABCD^	48.7 ± 1.6^ABCD^	1.7 ± 0.2^AB^		2.9 ± 0.2^CD^
CeTit-Tan-Ce-Cipro	32.6 ± 2.3^A^	13 ± 1.6^ABCD^	51.2 ± 1.7^ACE^	1.7 ± 0.3^AB^		1.5 ± 0.2^CE^

a Differences among groups were first evaluated using one-way ANOVA, and pairwise comparisons for the indicated groups were performed using Tukey’s post hoc test. A indicates statistically significant differences (*p* < 0.05) relative to the Tit sample, B relative to NaTit, C relative to CeTit, D relative to CeTit-Tan and E relative to CeTit-Tan-Ce.

**Figure 4:**
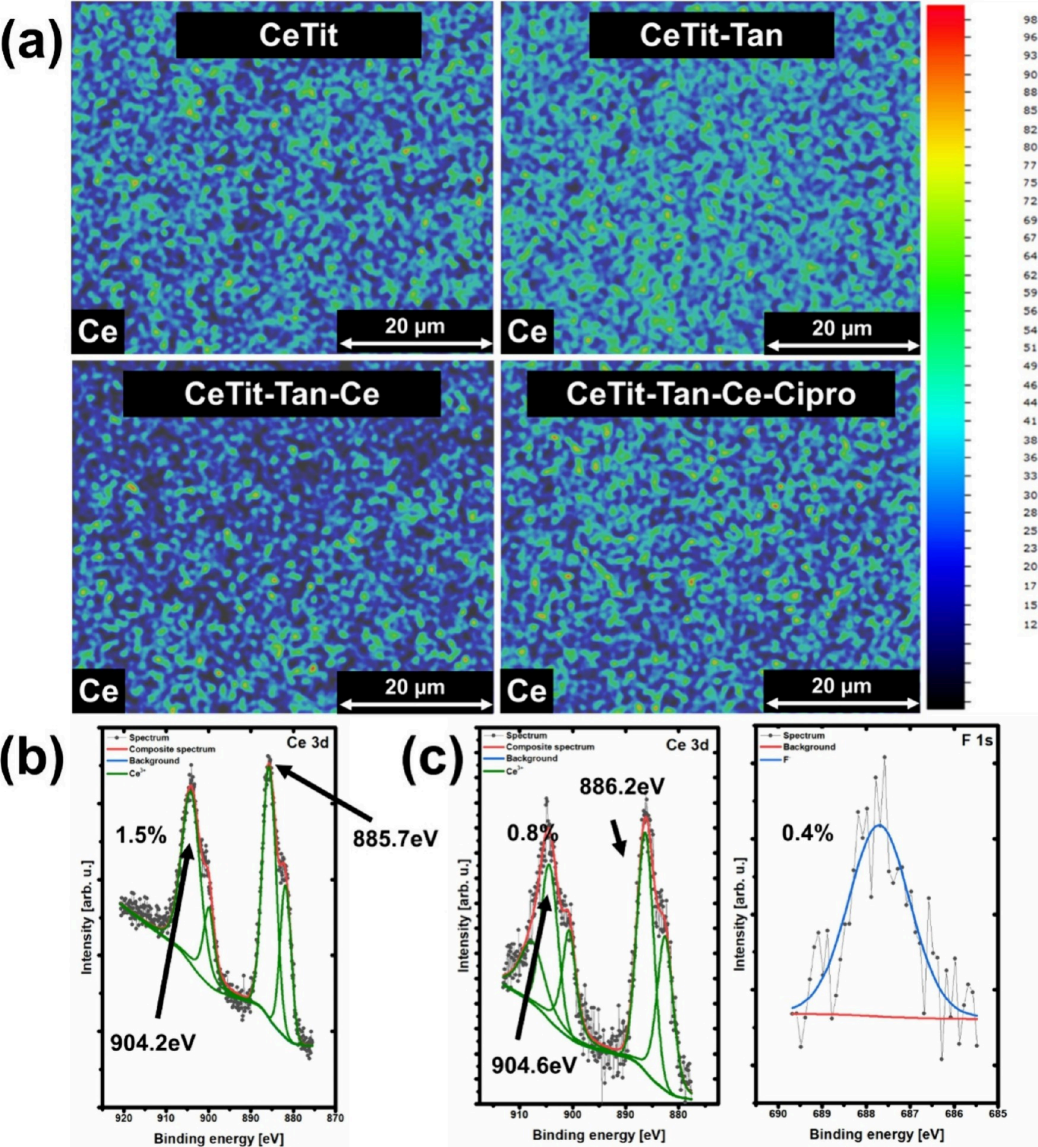
EDS mapping of cerium distribution in selected samples (a), high resolution XPS spectra of Ce 3d of the CeTit-Tan-Ce sample (b), and high resolution XPS spectra of Ce 3d and F 1s of the CeTit-Tan-Ce-Cipro sample (c).

To confirm the effectiveness of all stages of the proposed modification, the CeTit-Tan-Ce and CeTit-Tan-Ce-Cipro samples were analyzed using XPS. Elemental composition and high-resolution XPS spectra for Ti, C, O, N, Ce, and F elements are presented in Figures 4b,c and S1–S2. As can be observed, 6.1% of Ti^4+^ is present in the CeTit-Tan-Ce sample. The presence of this type of ion is typical for the titanate layer.^35^ The XPS measurement probes a depth of approximately 5–10 nm.^36^ The presence of titanium, therefore, indirectly indicates that the obtained cerium-loaded polyphenol layer has a thickness of less than 10 nm. The C 1s spectra for both samples were fitted with four components. The first peak, centered at 285.0 eV, arises from aliphatic carbon C–C bonds. The second peak, located at 286.4 eV, indicates the presence of C–O and/or C–OH and/or C–NH bonds. The third peak, centered at 287.9 eV, suggests the presence of C=O and/or O–C–O bonds,^37,38^ while the fourth peak, at 290.0 eV, indicates the presence of O–C=O type compounds.^37^ All of these bonds can be observed in the structure of tannic acid and ciprofloxacin. It can also be observed that the carbon content of the sample increases, suggesting surface adsorption of ciprofloxacin. The N 1s spectrum of both shows two peaks. These can be attributed to C–N amine-type bonds and the NH^4+^ cation, respectively.^37,38^ In the CeTit-Tan-Ce sample, the presence of these bonds may be attributed to residual NH_4_^+^ ions remaining from APS during synthesis, while the C–N bonds could originate from residual TRIS buffer, which served as the reaction medium. After loading ciprofloxacin, an increase in nitrogen content from 1.4 to 2.7% is observed, along with a noticeable increase in the intensity of the C–N bond peak, which is present in the structure of ciprofloxacin. The O 1s spectra were fitted with two or three components. In both cases, the spectra show peaks indicating the presence of O–Me bonds on the surface. Other bonds identified in both samples include O=C, O–C, and O–Ti.^35,39,40^ Finally, the Ce 3d spectra revealed the presence of Ce^3+^ ions on the material’s surface. The percentage content of cerium ions obtained using EDS and XPS may differ due to the fact that XPS is a more surface-sensitive technique. As can be seen in Figure 4b,c, the peaks indicating its presence are shifted by 0.4 and 0.5 eV, respectively. Such shifts toward higher binding energies from 904.2 to 904.6 eV and from 885.7 to 886.2 eV indicate an interaction between cerium and ciprofloxacin, suggesting the formation of a coordination bond.^41^ Additionally, XPS analysis also confirmed the presence of fluorine on the surface. The F 1s spectrum was fitted with a single peak centered at 687.6 eV, which is typical for fluoroquinolone-based antibiotics.^42^ The presented results confirm the effective formation of a hybrid titanate/polyphenol nanostructured coating loaded with Ce^3+^ ions and coordinated ciprofloxacin. To complement the XPS results, molecular modeling studies were also performed, demonstrating that interactions between tannic acid, Ce^3+^ ions, and ciprofloxacin are feasible; these interactions are described in detail in the Supporting Information (Figure S3).

The next analysis performed was FT-IR spectroscopy. The FT-IR spectra are presented in Figure 5a,b. The unmodified titanium alloy does not exhibit any characteristic bands. After the formation of the cerium titanate layer, two new bands appear at wavenumbers 1631 and 1425 cm^–1^. These bands can be attributed to the vibrations of water molecules remaining on the surface of the material and the vibrations of CO_2_ adsorbed from the air, interacting with cerium ions, respectively.^43^ After the formation of the hybrid layer, additional new bands appear, which can be assigned to tannic acid. Specifically, bands at 1710 cm^–1^ and 1065 cm^–1^ are observed, corresponding to C=O stretching vibrations and −C–O vibrations, respectively.^44,45^ Further modifications by loading the material with cerium ions lead to additional spectral changes. For instance, in the range of approximately 1470–1300 cm^–1^, several new bands emerge. Additionally, a band at 1207 cm^–1^, corresponding to C–O vibrations, becomes visible. It is also noteworthy that the band at 1710 cm^–1^, associated with the vibrations of C=O groups, shows a significant increase in intensity after cerium ion adsorption, which may indicate interactions with Ce^3+^ ions. Similar behavior has been observed in other studies, where interactions between Ce^3+^ ions and tannic acid were reported.^46^ After the sorption of Cipro onto the prepared hybrid titanate-polyphenol nanostructured coating, further changes in the FT-IR spectra can be observed. New bands appear in the range of 1510–1450 cm^–1^. The band at 1490 cm^–1^ can be attributed to the vibrations of CH_2_ groups in the aromatic ring. Additionally, a band at 1575 cm^–1^ becomes visible. The band at 1270 cm^–1^ also exhibits increased intensity and can be assigned to the vibrations of the C–O bond bending.^47^ FT-IR microscopy was also used to assess the distribution of the polyphenol layer and ciprofloxacin on the surface of the prepared materials. These results are shown in Figure 5c,d. To determine the distribution of the poly­(tannic acid) layer, mapping was performed for the band in the range of 1770–1682 cm^–1^. As observed, the polyphenol layer is evenly distributed across the entire surface of the material. Furthermore, to evaluate the distribution of Cipro, mapping was conducted in the range of 1285–1255 cm^–1^. The results indicate that the drug is present across the entire surface. While certain areas contain slightly higher concentrations of the drug, the differences are minimal when considering the scale of the analysis.

**Figure 5:**
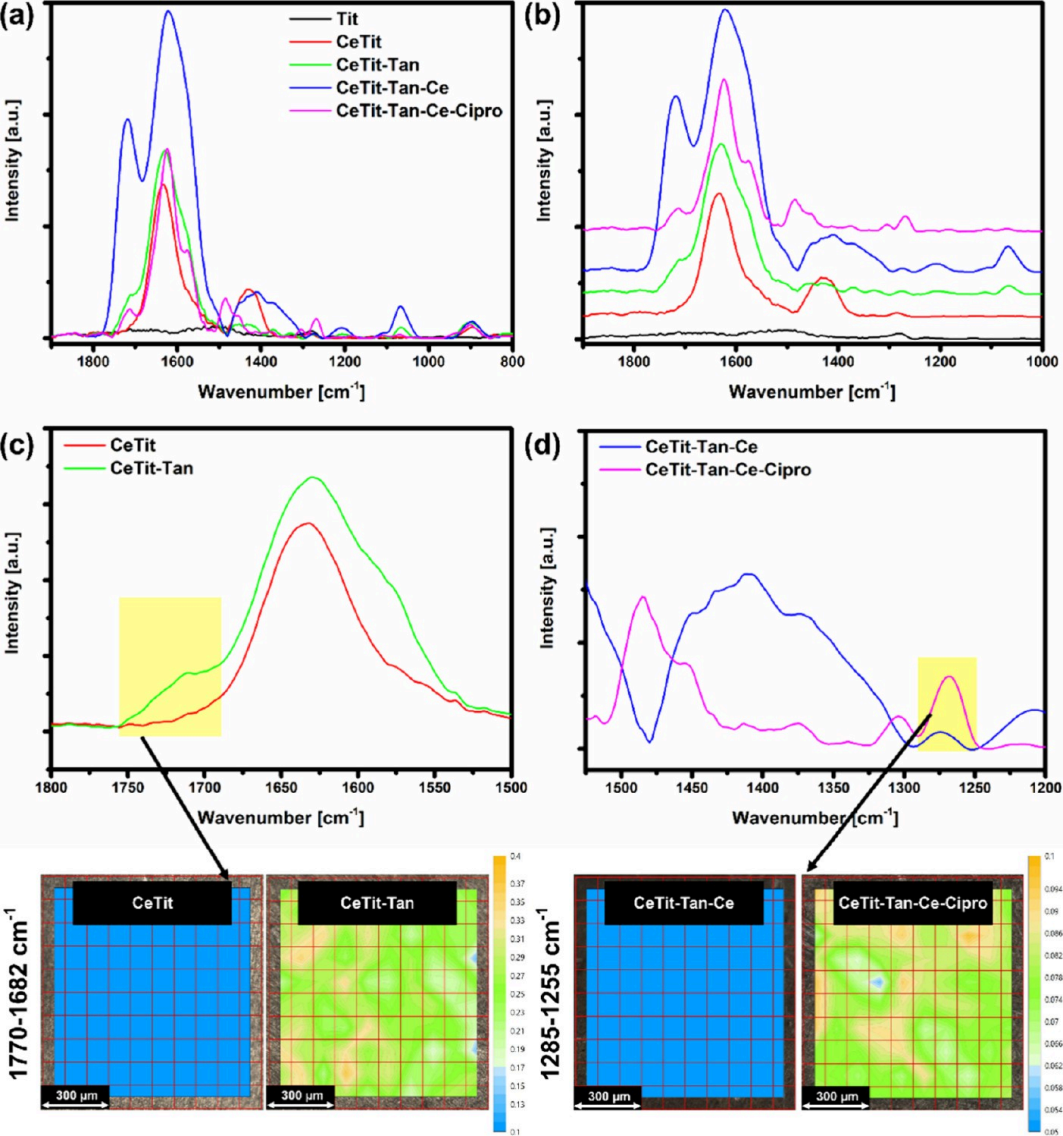
FT-IR spectra of the prepared materials: (a) overlaid spectra, (b) separated spectra to highlight changes, (c) zoomed-in region (1800–1500 cm^–1^) with mapping of the polyphenol layer distribution, (d) zoomed-in region (1550–1200 cm^–1^) with mapping of the drug distribution.

Another important aspect of the surface properties of biomaterials is their hydrophilicity. It affects, among other things, the ability of cells to adhere to the surface of implants.^48^ As shown in Table 4, the hydrophilicity of the material changed after each stage of modification. The unmodified titanium alloy had a contact angle of 80°, which is a typical value. After the formation of the titanate layer, the hydrophilicity significantly increased (contact angle around 50°). After ion exchange, the hydrophilicity increased slightly (the difference is statistically significant), and then, after obtaining the hybrid layer, the contact angle increased to around 60°. This is caused by the presence of hydrophobic aromatic rings in the structure of tannic acid. The most hydrophobic material obtained in this study was the sample labeled CeTit-Tan-Ce, with a contact angle of 98.78° ± 9.98°. A similar increase in the hydrophobicity of a tannic acid–based material loaded with Ce^3+^ ions was observed in the study by Haddadi et al.^46^ Subsequently, after the sorption of ciprofloxacin, the contact angle decreased to approximately 60°, which is considered an appropriate value for biomaterials.^48^ Additionally, to illustrate the obtained results in Figure 6a, images of the droplets formed during the WCA measurements for the prepared materials at 1 and 30 s are also presented.

**Table 4:** Results of Water Contact Angle Measurements^a^

sample name	WCA [°] after 1 s	WCA [°] after 30 s
Ti	80.4 ± 4.9	77.2 ± 5.4
NaTit	47.7 ± 5.4^A^	39.6 ± 5.3^A^
CeTit	32.6 ± 4.3^AB^	29.0 ± 4.3^A^
CeTit-Tan	57.5 ± 4.3^AC^	48.3 ± 7.2^AC^
CeTit-Tan-Ce	98.8 ± 10^ABCD^	90.1 ± 9.8^ABCD^
CeTit-Tan-Ce-Cipro	56.4 ± 4.3^ACE^	47.7 ± 5.1^ACE^

a The same letters within each column indicate no statistically significant differences. Differences among groups were first evaluated using one-way ANOVA, and pairwise comparisons for the indicated groups were performed using Tukey’s post hoc test. A indicates statistically significant differences relative to the Tit sample, B relative to NaTit, C relative to CeTit, and D relative to CeTit-Tan and E relative to CeTit-Tan-Ce-Cipro.

**Figure 6:**
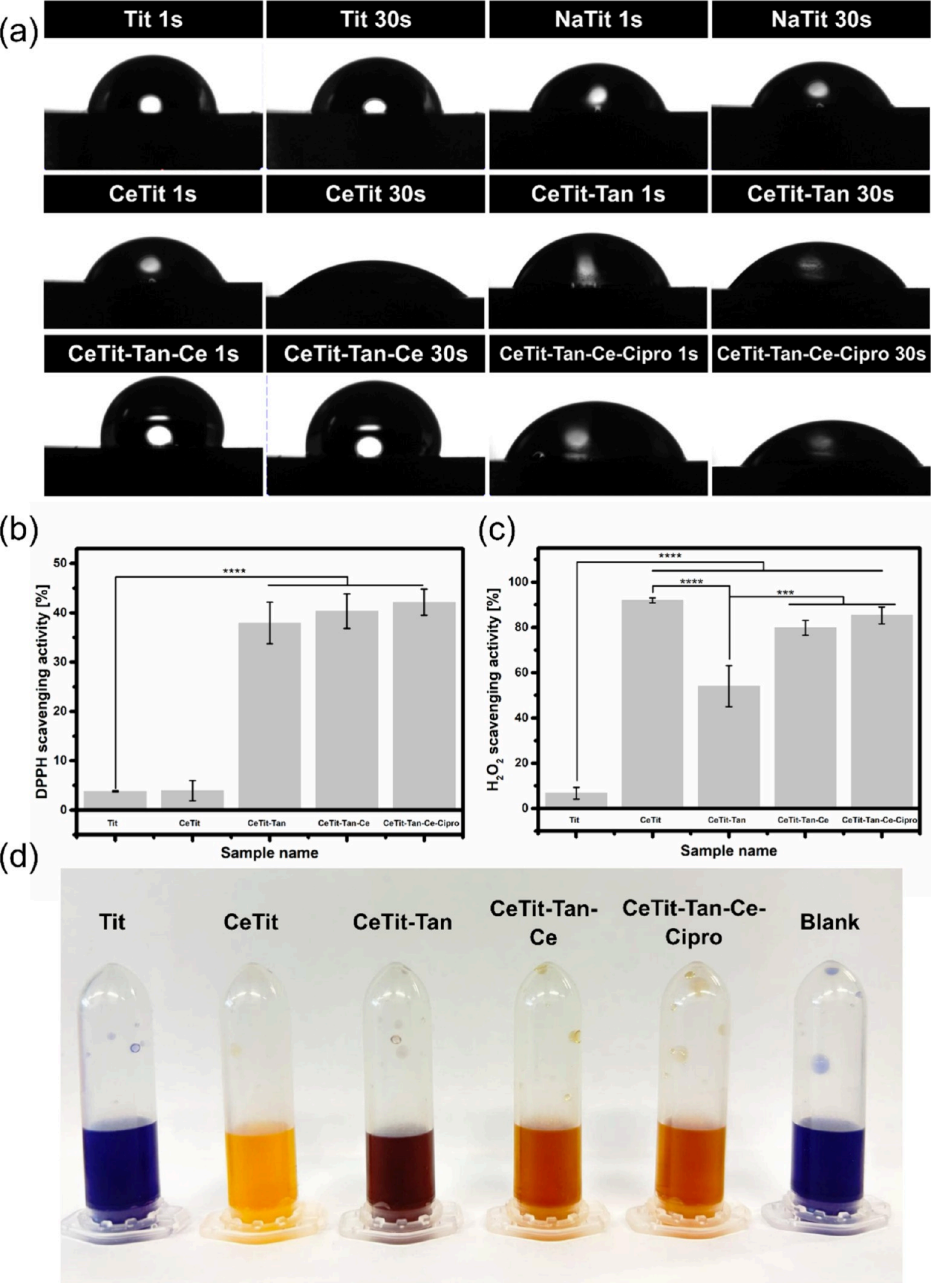
Images of the water contact angles of the prepared materials (a), DPPH scavenging activity assay (b), H_2_O_2_ scavenging activity assay (c), image of the samples after colorimetric reaction with H_2_O_2_ detection kit (the purple color indicates a high concentration of H_2_O_2_, while the yellow color indicates a low concentration of H_2_O_2_) (d). **** *p*-value <0.0001, ***p < 0.0005.

An additional advantage of titanium modification may be its antioxidant capability. Examples of reactive oxygen species (ROS) present in the human body include free radicals and hydrogen peroxide (H_2_O_2_).^6^ Polyphenols are well-known for their ability to stabilize free radicals; therefore, a DPPH scavenging activity test was conducted.^49^ As observed in Figure 6b, the Tit and CeTit samples exhibit negligible ability to stabilize DPPH. However, after modification with a polytannic acid layer, the sample was able to neutralize approximately 40% of the total DPPH content, which is an expected effect. The differences among the CeTit-Tan, CeTit-Tan-Ce, and CeTit-Tan-Ce-Cipro samples are statistically nonsignificant, indicating that they all exhibit similar DPPH scavenging activity. It is well documented that tannic acid is highly effective in neutralizing free radicals such as DPPH. This antioxidant activity results from the transfer of a hydrogen atom from a phenolic group to the DPPH free radical. The obtained tannic acid–based coating contains phenolic groups within its structure, enabling it to participate in the same reaction.^50^ It has been reported that Ce^3+^ may exhibit catalase-like activity, resulting in the ability to decompose H_2_O_2_. The mechanism of this process is based on the unique redox properties of the Ce^3+^/Ce^4+^ system. Specifically, cerium ions in the +3 oxidation state participate in the reaction:

$$2{\mathrm{Ce}}^{3+}+H_{2}O_{2}+2H^{+}\to 2H_{2}O+2{\mathrm{Ce}}^{4+}$$

and subsequently, cerium ions in the +4 oxidation state undergo the reaction

$$2{\mathrm{Ce}}^{4+}+H_{2}O_{2}\to 2{\mathrm{Ce}}^{3+}+O_{2}+2H^{+}$$

Thanks to these unique properties, materials containing cerium ions in their structure exhibit antioxidant activity.^51^ For this reason, the ability of the samples to decompose H_2_O_2_ was also investigated. The results are presented in Figure 6c,d. As observed, pure titanium is unable to decompose hydrogen peroxide. However, this changes significantly after the formation of the cerium titanate layer. The CeTit sample exhibits the greatest ability to decompose H_2_O_2_, but this ability significantly decreases (from approximately 90 to 50%, *p* < 0.0005) after modification with a polyphenol layer, most likely due to restricted access to Ce^3+^ ions. Nevertheless, this ability increases again to approximately 80% after additional loading of the layer with Ce^3+^ ions. Therefore, it can be concluded that cerium ions are responsible for the decomposition of H_2_O_2_ while tannic acid is responsible for a scavenger activity.^52^ Overall, this behavior demonstrates that the hybrid nanostructured coating enables the development of a material with the ability to stabilize both free radicals and hydrogen peroxide. Potentially allowing for a reduction of the body’s inflammatory response and the acceleration of osteogenesis.

One of the issues concerning implants, particularly hip implants, is the difference in Young’s modulus values between the material of the implant and the bone. For example, the Young’s modulus of the Ti_6_Al_4_V alloy is approximately 110 GPa, while bone exhibits a Young’s modulus in the range of 5–30 GPa. This difference causes the implant to bear the entire load, which can lead to bone resorption. One method to address this issue is the development of materials with a gradually changing Young’s modulus, which facilitates stress transfer between the implant and the bone.^53^ For this reason, nanomechanical studies were conducted to determine the Young’s modulus of layers at different stages of modification. As shown in Table 5, the formation of titanate layers results in a decrease in Young’s modulus to approximately 70 GPa. The addition of a polyphenol layer does not significantly affect the Young’s modulus (*p* > 0.05); however, the introduction of cerium ions leads to a reduction in Young’s modulus to around 40 GPa. Further addition of ciprofloxacin does not have a significant impact on this parameter (*p* > 0.05). By obtaining layers in which successive components exhibit a decreasing Young’s modulus, it may be possible to develop a material with improved stress transfer capabilities and reduced stress shielding effect.

**Table 5:** Parameters Were Determined Using the Nanoindentation Test^a^

sample name	hardness [GPa]	Young’s modulus [GPa]
Ti	1.69 ± 0.48	103.06 ± 15.37
NaTit	0.88 ± 0.20^A^	78.45 ± 8.10^A^
CeTit	0.61 ± 0.09^AB^	71.11 ± 6.66^A^
CeTit-Tan	0.65 ± 0.15^AB^	70.94 ± 7.46^A^
CeTit-Tan-Ce	0.45 ± 0.05^AB^	44.17 ± 2.40^ABCD^
CeTit-Tan-Ce-Cipro	0.35 ± 0.07^ABCD^	42.29 ± 4.04^ABCD^

a Differences among groups were first evaluated using one-way ANOVA, and pairwise comparisons for the indicated groups were performed using Tukey’s post hoc test. A indicates statistically significant differences (*p* < 0.05), relative to the Tit sample, B relative to NaTit, C relative to CeTit, and D relative to CeTit-Tan.

### 3.2 Drug Sorption and Release

A quantitative analysis of the sorption of the active substance was conducted using UV–vis spectroscopy. As shown in Figure 7, a plate with a geometric surface area of 0.8 cm^2^ was capable of retaining nearly 6 μg of Cipro. At this point, it is important to remember that the surface area of the actual implant is significantly larger, meaning that the real implant will contain a much greater amount of the drug on its surface. Additionally, the amount of loaded active substance must not be too high to avoid toxic effects. One may wonder whether the amount of antibiotic loaded into the material is sufficient. Based on literature sources, the minimum inhibitory concentration (MIC) of ciprofloxacin against *P. aeruginosa* is approximately 0.0625 μg/mL.^54^ For *S. aureus*, depending on the strain, reported MIC values range from 0.3 to 1.25 μg/mL.^55^ These values indicate that ciprofloxacin is a potent antibiotic capable of inhibiting bacterial growth at very low concentrations. As mentioned above, a real implant would possess a much larger surface area, allowing for the loading of a significantly greater total amount of the drug. Therefore, it can be concluded that the loaded drug dose should be sufficient for effective antibacterial activity. We can also observe that the CeTit-Tan sample was able to retain only about 2.5 μg of ciprofloxacin on the same surface area. The sorption of ciprofloxacin on this material may result from interactions with Ce^3+^ ions present in the cerium-titanate layer, as well as from hydrogen bonding or π–π interactions between ciprofloxacin and the polyphenolic portion of the coating. These results indicate that the additional step of loading the polyphenol layer with cerium ions enabled approximately twice the amount of drug to be retained, which indirectly supports the occurrence of coordination interactions between catechol groups in tannic acid cerium ions and ciprofloxacin. Regarding the release (also shown in Figure 7, it lasted for 34 h. As can be seen from the graph, more than 50% of the total retained ciprofloxacin was released within the first hour. The remaining amount was released over the next 34 h. This release profile ensures a therapeutic drug concentration immediately after implantation, while the remaining amount helps prevent infection within the first day after surgery. Similar release profiles were obtained for drug release in both pure SBF and SBF supplemented with HSA. However, lower amounts of the released drug were observed in the presence of HSA. This may be attributed to the adsorption of HSA onto the sample surface, which acts as a barrier and slows down the release of the active substance. We attempted to fit the release kinetics to existing kinetic models; however, none of them provided a satisfactory fit. This may be due to the fact that most classical release models are based on drug diffusion from a polymeric matrix, whereas in our system the drug is retained directly on the surface rather than embedded within a bulk material. Additionally, in one of our previous studies, we observed that the release of ciprofloxacin can be correlated with the strength of complexation between the drug and a given ion, as determined using density functional theory calculations.^56^ For this reason, it can be concluded that the primary release mechanism in the present system is the breakdown of the ciprofloxacin–metal complexes through interactions with the components of the SBF solution.

**Figure 7:**
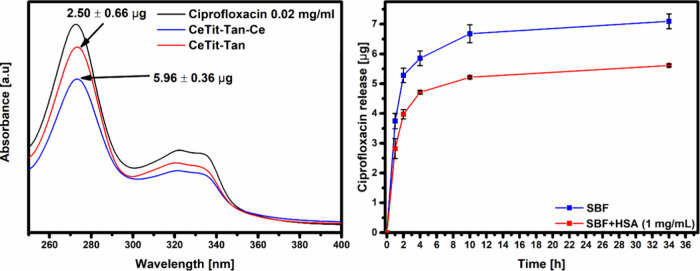
Amount of drug retained on the CeTit-Tan and CeTit-Tan-Ce samples (left) and the release profile of ciprofloxacin (right).

The obtained material cannot be easily compared to those currently available in the literature, as there are only a limited number of examples of polyphenol nanostructured coatings capable of releasing active substances. For example, in one study, titanium implants were modified with various polylactide-based copolymers and ciprofloxacin.^57^ The layers were prepared using the dip-coating technique. Despite the effective drug loading, layers obtained with this technique often exhibit poor mechanical properties and weak adhesion to the implant surface. In another study, the titanium alloy was modified with graphene oxide and levofloxacin.^58^ Levofloxacin is also a fluoroquinolone drug with a structure similar to Cipro. However, in that study, the drug was released for only 3 h. In contrast, this study achieved ciprofloxacin release for 34 h, which is a significantly longer result.

### 3.3 Effects of the Developed Materials on Cell Behavior

Assessing the cytotoxicity of a titanium-based implant is a fundamental step in determining its suitability for bone tissue integration, as it provides insight into its impact on cell viability. These studies are a critical component of biocompatibility assessment, which determines the safe and long-term performance of the implant within the biological environment.^59^ According to ISO 10993–5 standard, none of the tested samples was toxic (Figure 8a). The only significant difference compared to the control existed in CeTit sample (modification with cerium), where the cell viability was about 90%. Nevertheless, the mentioned sample should be still considered as nontoxic since the ISO standard indicates material as safe and noncytotoxic if cell viability after incubation with material extract is above 70% compared to the control cells.

**Figure 8:**
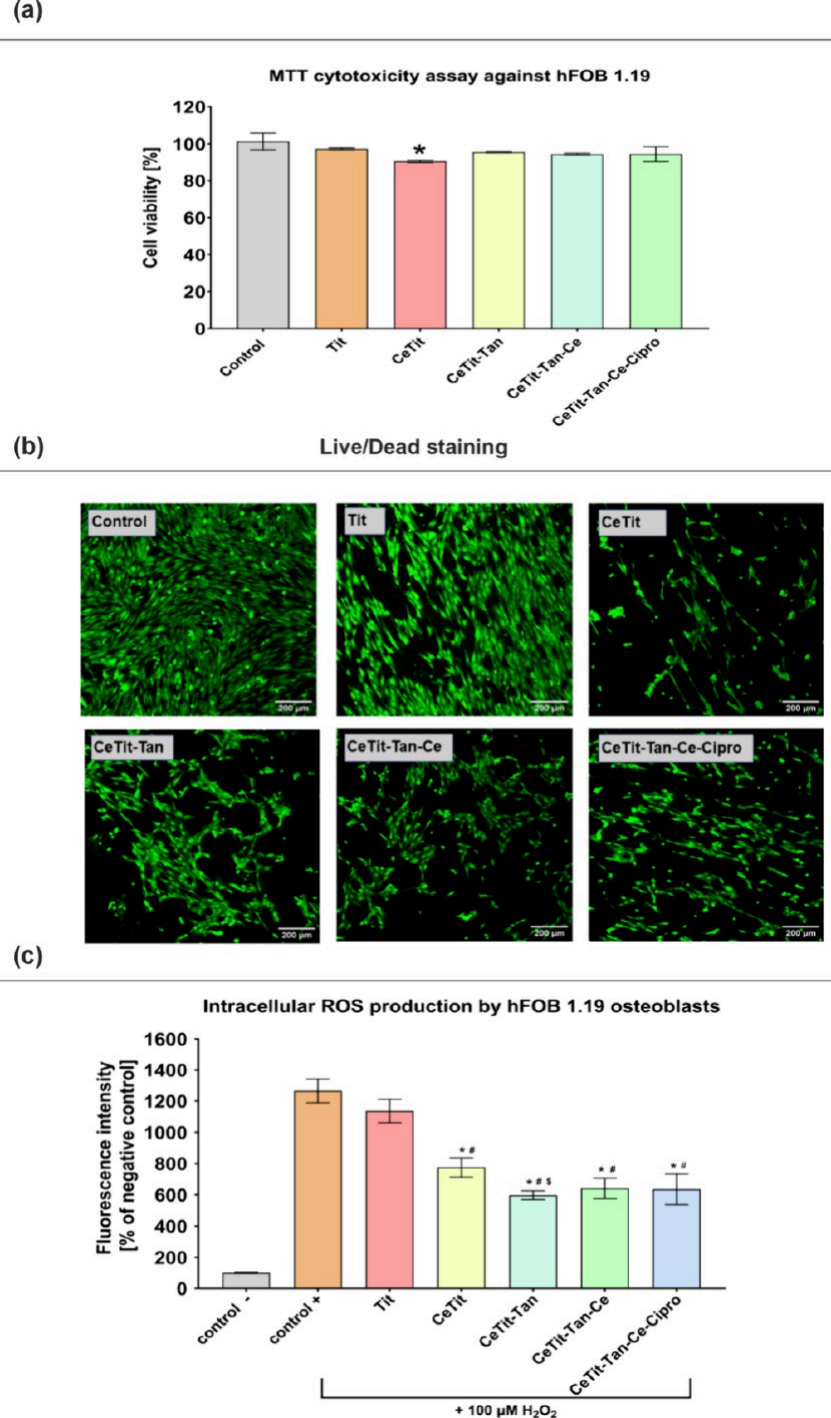
In vitro cell culture experiments: (a) MTT cytotoxicity assay conducted after 24 h (control — cell treated with polystyrene extract), * statistically significant results compared to the control (*p* < 0.05, one-way ANOVA followed by Tukey post hoc test) (b) CLSM images of osteoblasts stained with Live/Dead kit after 72-h culture on the materials (green fluorescence – viable cells, red fluorescence – dead cells; scale bar = 200 μm), (c) ability of the samples to reduce intracellular ROS production by osteoblasts activated with H_2_O_2_; * significantly different results compared to the positive control (cells cultured without tested materials), # significantly different results compared to the Tit sample, $ significantly different results compared to the CeTit sample (*p* < 0.05, one-way ANOVA followed by Tukey post hoc test).

Live/Dead staining (direct cytotoxicity test) performed 72 h after seeding the cells on the material confirmed that all tested samples were nontoxic, supported cell adhesion on their surfaces, and maintained normal osteoblast morphology. No dead cells (indicated by red-stained nuclei) were observed. Compared to the pure titanium sample, fewer cells were observed on the modified biomaterials, which may indicate that additional modifications slightly hindered cell adhesion (Figure 8b), as usual on rough surfaces which promote osseodifferentiation. An important observation in this study is that the Live/Dead assay did not reveal any growth reduction on the antibiotic-coated surface compared to other modified variants. While the incorporation of ciprofloxacin as a surface coating on titanium-based bone implants raises valid concerns regarding potential cytotoxic effects and the development of bacterial resistance, it has nonetheless been explored as a preventive strategy against postsurgical complications. Notably, such an approach may effectively reduce the risk of bacterial colonization and infection in the implant region, particularly during the early postimplantation period.^60^

Evaluation of intracellular ROS production by osteoblasts activated with H_2_O_2_ clearly showed that the samples loaded with cerium ions and tannic acid had evident antioxidant activity (Figure 8c). The antioxidant potential of cerium-modified samples is probably due to the presence of cerium capable of cyclic redox transformations Ce^3+^/Ce^4+^, enabling catalytic neutralization of ROS, including H_2_O_2._ ^61^ Tannic acid, thanks to its polyphenolic nature, has the ability to chelate metal ions, stabilizing the material surface ions. Additionally, antioxidant potential of tannic acid is also related to the presence of numerous hydroxyl groups in its structure.^62,63^ Overall, it can be concluded that the obtained samples exhibit excellent antioxidant properties, which are not compromised by the incorporation of ciprofloxacin into the material.

### 3.4 Antibacterial Properties

The microorganisms most frequently isolated from infected implantation sites are *Pseudomonas aeruginosa* and *Staphylococcus aureus*.^64^ It is particularly important to protect the implant when it is most vulnerable to microbial contamination, i.e., during the surgical procedure.^65^ Prevention of bacterial infections of bone implants includes coating the biomaterial with various antibacterial compounds.^66^

The results of the direct contact antibacterial test showed no statistically significant decrease in the density of the bacterial suspension after exposure to a titanium surface and its modifications with Ce^3+^ ions and tannic acid (Figure 9a).

**Figure 9:**
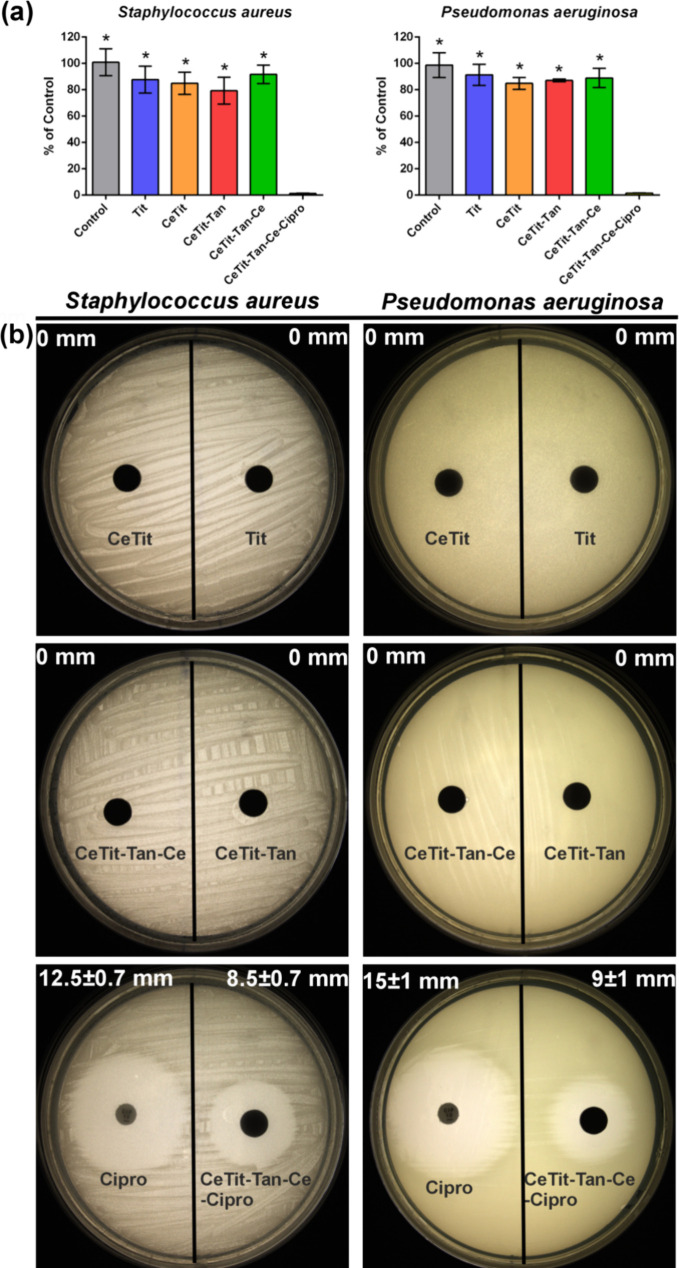
(a) Evaluation of bacterial growth inhibition in direct contact with the implant, presented as % of positive control (bacteria grown in M-H broth without material),* statistically significant results compared to CeTit-Tan-Ce-Cipro material, *p*-value <0.05, one-way ANOVA followed by Tukey’s test; (b) images of growth inhibition zones obtained with agar diffusion test (ciprofloxacin disk served as reference control).

However, loading of the antibiotic on the implant surface in the CeTit-Tan-Ce-Cipro material resulted in almost complete elimination of both types of bacteria from the M-H broth. A decrease in bacterial culture density of 98.4% was noted for *P. aeruginosa* and 98.8% for *S. aureus*. The results of the direct contact experiment were confirmed by the agar diffusion test. No growth inhibition zone was observed after using the following materials: Ti, CeTit, CeTit-Tan, or CeTit-Tan-Ce (Figure 9b). Nevertheless, using the CeTit-Tan-Ce-Cipro disk resulted in growth inhibition zones of 9.0 ± 1.0 and 8.5 ± 0.7 mm for *P. aeruginosa* and *S. aureus*, respectively (Figure 9). After application of the ciprofloxacin reference disk, a growth inhibition zones of 15.0 ± 1.0 and 12.5 ± 0.7 mm for *P. aeruginosa* and *S. aureus*, respectively were observed (Figure 9b). The differences in growth inhibition zones between the tested sample and the reference antibiotic disk resulted from the divergent ciprofloxacin content. The drug sorption and release results showed that the CeTit-Tan-Ce-Cipro disk with a surface area of 0.385 cm^2^ contained 2.89 μg of antibiotic, while the reference disk with a surface area of 0.283 cm^2^ contained significantly greater amount of antibiotic −10 μg. It is worth noting that despite the 3.5-fold difference in antibiotic content, the growth inhibition zones between the biomaterial and the reference antibiotic disk differed only about 1.7–1.5-fold. Interestingly, the reference antibiotic disk demonstrated higher activity against Gram-negative bacteria compared to Gram-positive bacteria, as also indicated by literature data. However, the antibacterial activity of the CeTit-Tan-Ce-Cipro biomaterial against *S. aureus* and *P. aeruginosa* was almost identical, indicating equally good antibiotic penetration into bacterial cells of varying cell wall thickness and architecture.

Biofilms are a critical factor in the persistence of bacteria on the surface of bone implants. Bacteria embedded in biofilm structures are much more resistant to therapeutic interventions than their planktonic forms.^67^ Since only the sample loaded with Cipro showed antibacterial properties, antibiofilm activity was evaluated for the control surface (polystyrene), unmodified Tit sample, and CeTit-Tan-Ce-Cipro material. The CeTit-Tan-Ce-Cipro sample demonstrated a significant inhibitory effect on the adhesion of both Gram-positive *S. aureus* and Gram-negative *P. aeruginosa*, with a more pronounced effect observed for the Gram-negative strain (Figure 10). Biofilms formed on the CeTit-Tan-Ce-Cipro surface were characterized by a predominance of dead red-fluorescent cells, whereas viable green-fluorescent bacteria of both strains were present at much lower density. In contrast, the titanium surface supported a high density of viable bacterial cells, however, the resulting bacterial layer was less dense and less compact than that observed on the polystyrene control.

**Figure 10:**
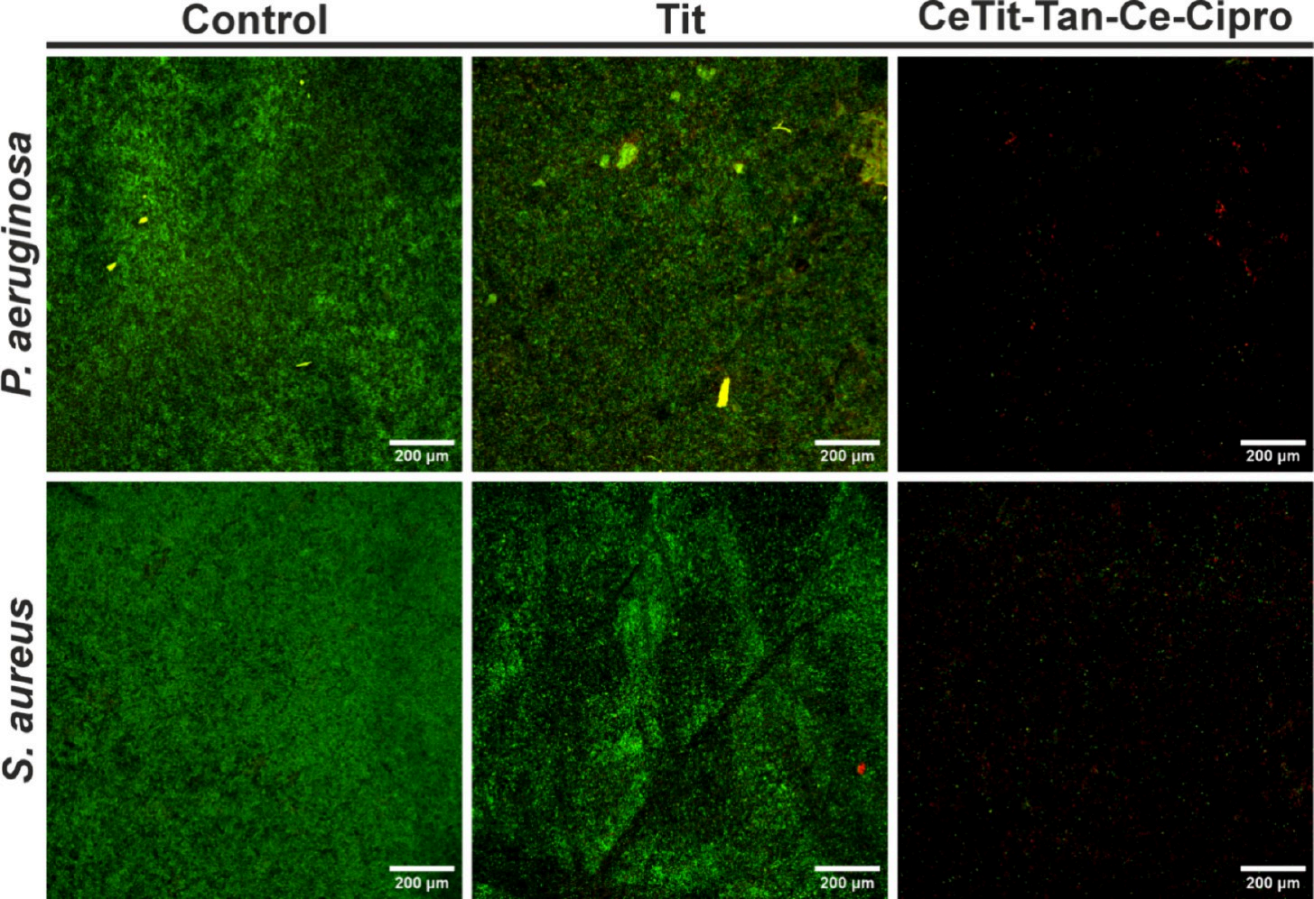
Images obtained from a confocal microscope showing the biofilm structure of *S. aureus* and *P. aeruginosa* bacteria on the surface of analyzed biomaterials, imaged using Live/Dead fluorescent staining; bacterial biofilm growing on polystyrene was used as a control (100× magnification, 200 μm scale).

The study results indicated that the CeTit-Tan-Ce-Cipro material, due to the incorporation of the antibiotic, effectively reduced not only the viability of planktonic bacteria but also bacterial adhesion and survival within the biofilm structure. Ciprofloxacin is a member of the fluoroquinolone class of antibiotics and exhibits broad-spectrum antibacterial activity. Its primary molecular target is bacterial DNA. The mechanism of its action involves inhibition of DNA gyrase activity, an enzyme composed of A and B subunits. Ciprofloxacin interferes with the DNA religation process of double-stranded DNA mediated by the A subunit, making single-stranded DNA susceptible to exonucleolytic degradation.^68,69^ In other studies conducted by our research team, a titanium material also modified with polyphenols (*epigallocatechin gallate*) and Zn^2+^ ions was created. In microbiological tests on *S. aureus* and *P. aeruginosa* bacteria, similar antibacterial activity was observed to the studies presented in this article, but with a higher antibiotic content in the biomaterial.^70^ Zhou et al. and Hajinaebi et al. also demonstrated high antibacterial activity against *S. aureus* and *E. coli* for titanium implants modified with ciprofloxacin.^71,72^

### 3.5 Comparison of the Prepared Coating with Ce-Containing Coatings Reported in the Literature

There are several studies describing the modification of implant surfaces using cerium-containing coatings. Most of these coatings focus on the use of cerium ions in the form of CeO_2_; however, other approaches have also been reported in the literature. A comparison of the coating prepared in this study with those reported in the literature is shown in Table 6. For example, one study utilized a cerium titanate layer as a carrier for the antibiotic tetracycline.^26^ In that work, the authors demonstrated the anticorrosive potential of the obtained coating. Regarding the antibiotic release duration, it lasted only 4 h, which is significantly shorter than the release time achieved in our study. Additionally, antibacterial tests showed that the material prepared in that work was capable of inhibiting only *S. aureus*, whereas in our study inhibitory activity was observed against both Gram-negative and Gram-positive bacteria. In another study, Priyadarshini et al. proposed modifying Ti_6_Al_4_V surfaces using Ce-doped hydroxyapatite (HAp) combined with Fe_3_O_4_ nanoparticles.^73^ Although this approach demonstrated good anticorrosive, antibacterial, and biocompatible properties, it has a notable limitation. Specifically, the coating in their study was applied using the spin-coating technique, which restricts its applicability. Implant surfaces often have complex geometries, whereas spin-coating is suitable mainly for flat substrates. In contrast, the methodology used in our work enables the fabrication of coatings on materials of virtually any shape. In another study, De Santis et al. performed a multistep modification of Ti and Ti_6_Al_4_V alloy surfaces.^74^ First, the material was anodized to produce TiO_2_ nanotubes. Subsequently, using a drop-casting approach, a cerium nitrate solution was applied to the surface, followed by annealing at 400 °C to form cerium oxides. This process was repeated several times. The resulting coatings exhibited a significant increase in corrosion resistance, even in the presence of H_2_O_2_, used as a model ROS. The materials obtained by the authors were biocompatible and showed some antibacterial activity; however, it should be noted that the antibacterial tests employed a very small volume of bacterial suspension (0.005 mL). In our study, the volume of bacterial suspension used was substantially higher, which further highlights the stronger antibacterial properties of the ciprofloxacin-loaded material. The last study discussed is the work by Yao et al.^75^ In this research, the authors modified a Ti-based implant using a layer-by-layer self-assembly technique. The coating components included carboxylated chitosan, nano-CeO_2_, and hyaluronic acid modified with phenylboronic acid. This combination enabled the formation of a coating capable of responding to external stimuli, specifically by reacting to increased levels of reactive oxygen species (ROS). The materials obtained in this study, containing CeO_2_ within their structure, exhibited excellent antioxidant activity under conditions of elevated oxidative stress, as evidenced by the increased expression of ROS-related genes. The CeO_2_-loaded samples also showed reduced levels of MDA, an indicator of oxidative damage. Additionally, the materials were capable of enhancing the expression of osteogenic genes such as alkaline phosphatase and COL1. Moreover, the coatings promoted accelerated in vivo osseointegration. However, the materials developed in that study did not demonstrate any confirmed antibacterial activity, which represents a significant advantage of the coating presented in our work.

**Table 6:** Comparison of the Properties of the Ce-Based Coating Developed in This Study with Ce-Containing Coatings Described in the Literature

type of modification	functionality	drug release	antibacterial activity	biocompatibility	reference
cerium titanate layer loaded with tetracycline	antibiotic release, anticorrosion activity, antibacterial activity	the release of tetracycline lasted for 240 min	growth inhibition of bacteria’s from *S. aureus* strain. Lack of activity against *E. coli* strain		^26^
Ce-doped hydroxyapatite/Fe_3_O_4_ composite spin coated on Ti_6_Al_4_V	anticorrosion activity, antibacterial activity		growth inhibition of bacteria from *E. coli, S. Aureus* and *P. aeruginosa* strains	noncytotoxic to Mg63 cell line	^73^
titanium nanotubes layer modified further with cerium oxides through drop-casting/annealing cycles	anticorrosive, biocompatible, antibacterial		antibacterial activity to *S. aureus* and *P. aeruginosa* strains	biocompatible to MRC5 human fibroblast	^74^
layer by layer self-assembly of carboxylated chitosan, modified hyaluronic acid and CeO_2_	biocompatible, enhanced expression of osteorelated genes, enhanced osteointegration, antioxidative activity			biocompatible to MC3T3-E1 cell line, good MC3T3-E1 cells adhesion, biocompatible in vivo in rats	^75^
cerium titanate - poly(tannic acid) – Ce^3+^ layer loaded with ciprofloxacin	biocompatible, antioxidative, antibacterial, sustained antibiotic release	the release of ciprofloxacin lasted for 34 h	antibacterial activity against S. *aureus* and *P. aeruginosa* in direct contact and agar diffusion assays	noncytotoxic to hFOB 1.19 cell line, the cells adhered well to the surface of modified materials	this work

## 4 Conclusions

The presented work demonstrates the modification of the Ti_6_Al_4_V titanium alloy surface with a hybrid nanostructured coating composed of cerium titanate and a poly­(tannic acid) layer. The coating was further reloaded with Ce^3+^ ions and the antibiotic ciprofloxacin. The effectiveness of each modification step, as well as drug loading, was confirmed using SEM, EDS, and XPS analyses. FT-IR microscopy revealed that both the poly­(tannic acid) layer and ciprofloxacin were uniformly distributed across the surface. XPS analysis allowed for confirmation of formation of coordination interactions between ciprofloxacin and Ce^3+^. The conducted studies showed that the material was capable of releasing ciprofloxacin for 34 h, exhibiting a sustained release profile both in SBF and SBF with addition of human serum albumin. An important feature of the obtained layer is its antioxidant activity, as it was able to scavenge both free radicals and H_2_O_2_. Biological evaluations demonstrated that the developed material was noncytotoxic and supported cell adhesion on its surface. The obtained materials also exhibited the ability to neutralize intracellular reactive oxygen species. Antibacterial tests revealed that ciprofloxacin-loaded samples exhibited significant antibacterial and antibiofilm activity against *Staphylococcus aureus* and *Pseudomonas aeruginosa*. We believe that the developed material holds strong promise as a functional coating for titanium implants, particularly dental implants, which are highly susceptible to bacterial colonization. The present study builds upon our earlier research and further demonstrates that titanium-based implants can be engineered as efficient drug-delivery platforms by incorporating polyphenols, antibiotics, and metal ions. Looking ahead, the performance of the proposed hybrid nanostructured coating may be further optimized through adjustments in synthesis parameters and processing conditions.

## Supplementary Material



## Data Availability

Data supporting the findings of this study are available in the open RepOD repository with the identifier 10.18150/Z2UNKI.

## Notes

High-resolution XPS spectra of Ti 2p, C 1s, N 1s, and O 1s for CeTit-Tan-Ce and CeTit-Tan-Ce-Cipro samples; molecular modeling details of Ce^3+^ coordination with ciprofloxacin and tannic acid; graphical representation of the optimized coordination model and calculated interaction energy (PDF)
