# Supplementary material for: Development of a Cerium-Doped Titanate/Polyphenol Nanostructured Coating for Titanium Implants: Enhancing Antibacterial Properties through Ciprofloxacin Release
Source: ACS Appl Mater Interfaces. 2026 Mar 17;18(12):17660–78. doi: 10.1021/acsami.6c03707 (PMC13298815; doi:10.1021/acsami.6c03707)
Supplement: Supplementary file 1 [file am6c03707_si_001.pdf]

Supporting Information

# Development of a Cerium-Doped Titanate/Polyphenol Nanostructured coating for Titanium Implants: Enhancing Antibacterial Properties through Ciprofloxacin Release

Marcel Jakubowski[1]*, Mateusz Hegmit[1], Maria Ratajczak[2], Marta Trzaskowska[3], Aleksandra Maciejczyk[3], Agata Przekora[3], Monika Zielińska[1], Łukasz Ławniczak[1], Silvia Spriano[4], Mariusz Sandomierski[1*]

[1] Institute of Chemical Technology and Engineering, Poznan University of Technology, ul. Berdychowo 4, 60-965 Poznań, Poland

[2] Institute of Building Engineering, Poznan University of Technology, ul. Piotrowo 5, 60-965 Poznań, Poland

[3] Department of Tissue Engineering and Regenerative Medicine, Medical University of Lublin, Chodzki 1, 20-093 Lublin, Poland

[4] DISAT Department, Politecnico di Torino, Corso Duca degli Abruzzi 24, 10129 TORINO, Italy

*e-mail: mariusz.sandomierski@put.poznan.pl

*e-mail: marcel.jakubowski@doctorate.put.poznan.pl

## 1. XPS analysis

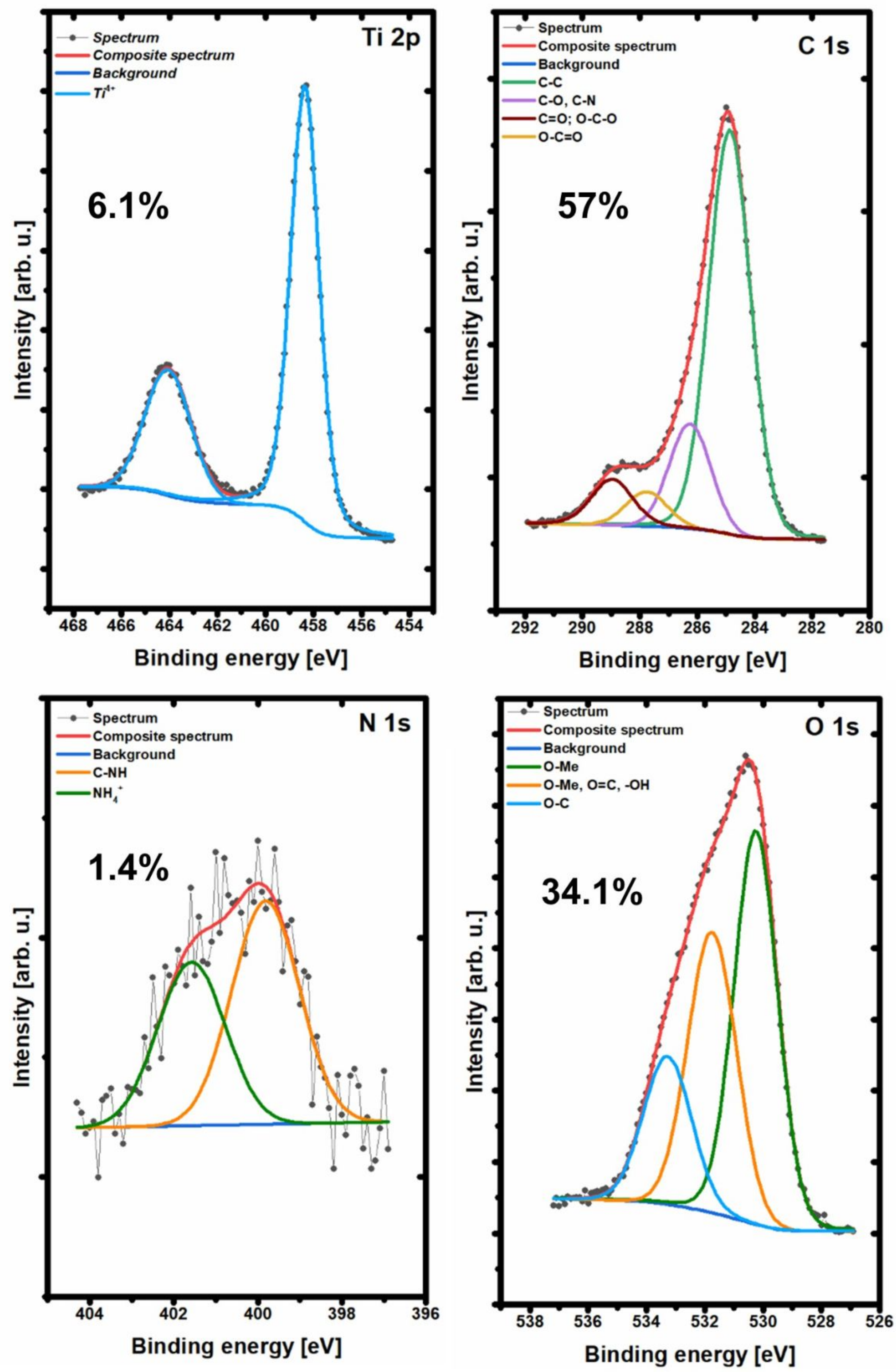


**Figure S1.** High resolution XPS spectra of Ti 2p, C 1s, N 1s and O 1s of the CeTit-Tan-Ce sample.

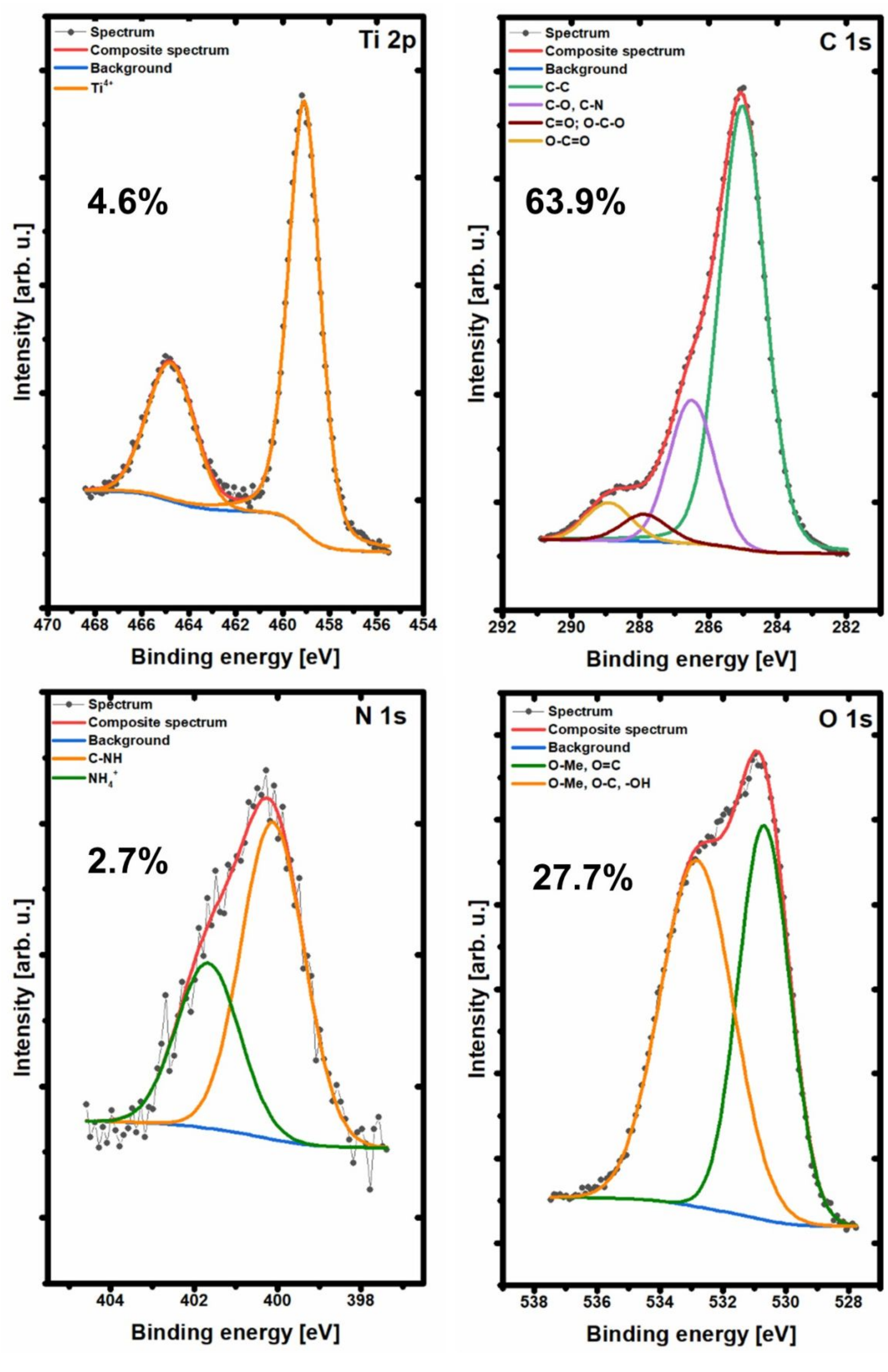


**Figure S2.** High resolution XPS spectra of Ti 2p, C 1s, N 1s and O 1s of the CeTit-Tan-Ce-Cipro sample.

## 2. Molecular modelling

Molecular modelling was utilized in order to provide additional theoretical background for the formation of metal-coordination bonds between $Ce^{3+}$ and Cipro. Each respective model ($Ce^{3+}$, ciprofloxacin and tannic acid) was prepared using the Hyperchem software and pre-optimized using the Gaussian software at the M06/def2TZVP level. Then, the models were combined into systems with different starting geometries, which were subsequently optimized and visualized using the GausView software. The relevant part of the optimized system is presented below:

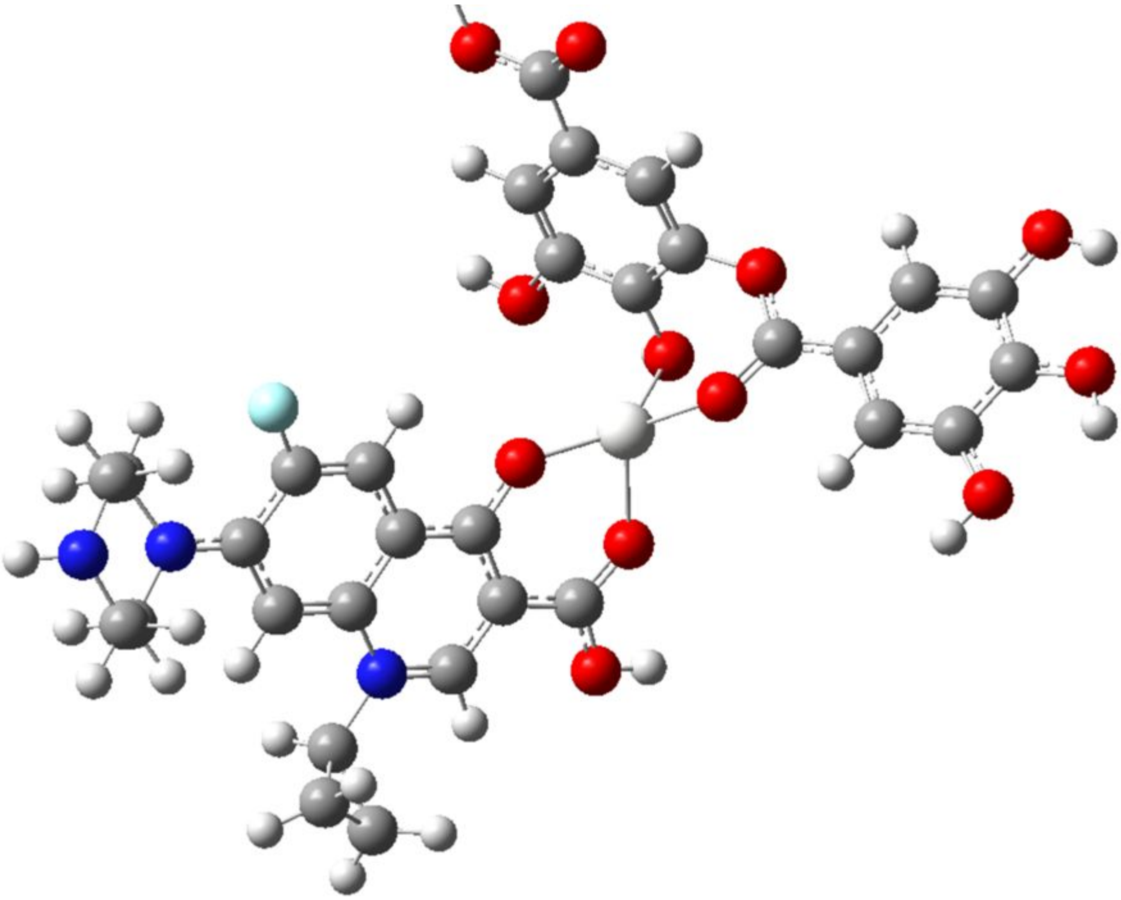

**Figure S3.** Graphical representation of the Tannic acid-$Ce^{3+}$-Cipro coordination model.

The model suggests that the cerium cation can be coordinated by both ciprofloxacin (by contribution of the carbonyl and carboxyl group) as well as tannic acid (in this case the hydroxyl and ester group are involved). The calculated interaction energy was equal to approx. -614 kcal/mol, which indicates strong binding. The value was obtained for a simple no-solvent model and is generally in accordance with values reported in case of gas-phase DFT calculations for lanthanide complexes, which can reach hundreds of kcal/mol [1]. This further supports the concept that $Ce^{3+}$ is capable of forming a metal-coordination bond with Cipro.

[1] R.D. O'Brien, T.J. Summers, D.S. Kaliakin, D.C. Cantu, The solution structures and relative stability constants of lanthanide–EDTA complexes predicted from

computation, Phys. Chem. Chem. Phys. 24 (2022) 10263–10271. https://doi.org/10.1039/D2CP01081J.
